# Predicting plant Rubisco kinetics from RbcL sequence data using machine learning

**DOI:** 10.1093/jxb/erac368

**Published:** 2022-09-12

**Authors:** Wasim A Iqbal, Alexei Lisitsa, Maxim V Kapralov

**Affiliations:** School of Natural and Environmental Sciences, Newcastle University, Newcastle upon Tyne, NE1 7RU, United Kingdom; Department of Computer Science, University of Liverpool, Liverpool, L69 3BX, United Kingdom; School of Natural and Environmental Sciences, Newcastle University, Newcastle upon Tyne, NE1 7RU, United Kingdom; Lancaster University, UK

**Keywords:** Enzyme, Gaussian process, kinetics, machine learning, photosynthesis, Rubisco

## Abstract

Ribulose-1,5-bisphosphate carboxylase/oxygenase (Rubisco) is responsible for the conversion of atmospheric CO_2_ to organic carbon during photosynthesis, and often acts as a rate limiting step in the later process. Screening the natural diversity of Rubisco kinetics is the main strategy used to find better Rubisco enzymes for crop engineering efforts. Here, we demonstrate the use of Gaussian processes (GPs), a family of Bayesian models, coupled with protein encoding schemes, for predicting Rubisco kinetics from Rubisco large subunit (RbcL) sequence data. GPs trained on published experimentally obtained Rubisco kinetic datasets were applied to over 9000 sequences encoding RbcL to predict Rubisco kinetic parameters. Notably, our predicted kinetic values were in agreement with known trends, e.g. higher carboxylation turnover rates (Kcat) for Rubisco enzymes from C_4_ or crassulacean acid metabolism (CAM) species, compared with those found in C_3_ species. This is the first study demonstrating machine learning approaches as a tool for screening and predicting Rubisco kinetics, which could be applied to other enzymes.

## Teaser

Machine learning approaches are used as a tool to predict Rubisco kinetics from RbcL sequences.

## Introduction

Ribulose-1,5-bisphosphate carboxylase/oxygenase (Rubisco) is claimed to be the most abundant enzyme on Earth (Bar-On and Milo, 2019). The global conversion of inorganic CO_2_ to organic forms is mostly driven by Rubisco, making it a gatekeeper of carbon for nearly all life on the planet (Raven, 2013). Form IB Rubisco proteins found in plants and green algae consist of both large and small subunits, and the large subunits contain the Rubisco active site. Thus, it has long been assumed that the large subunit sequence variation contributes to the diversity of Rubisco kinetics (Kellogg and Juliano, 1997; Camel and Zolla, 2021). Rubisco is often characterised as having a slow turnover rate (Kcat) for CO_2_ and poor specificity for CO_2_ compared with O_2_ (Sc/o; but see Tcherkez *et al.*, 2006). Rubisco catalytic inefficiencies might limit plant photosynthetic performance in certain environmental conditions such as saturating irradiance and limiting CO_2_ concentrations. Improving Rubisco kinetic traits is therefore a target for improving plant carbon uptake and crop yield. One strategy of doing this is screening the natural diversity of Rubisco kinetics and replacing native Rubisco enzymes in plants with catalytically more efficient enzymes (Ort *et al.*, 2015; Hermida-Carrera *et al.*, 2016; Orr *et al.*, 2016; Sharwood *et al.*, 2016; Galmés *et al.*, 2019; Orr and Parry, 2020; Von Caemmerer, 2020; Iqbal *et al.*, 2021; Johnson, 2022; Lin *et al.*, 2022). Although there has been some progress with this strategy, direct replacement of Rubisco in crops is currently challenging, due to both limited capacity to mass-screen Rubisco kinetics, and Rubisco chaperone incompatibilities between distant species (Kanevski *et al.*, 1999; Whitney *et al.*, 2011, 2015; Wilson *et al.*, 2016, 2018; Sharwood, 2017; Zhou and Whitney, 2019; Gunn *et al.*, 2020; Martin-Avila *et al.*, 2020).

Given the resource-intensive nature of screening enzyme kinetics in the laboratory, modelling or *in silico* approaches, such as machine learning (ML), are being increasingly adopted to aid bioengineering efforts (Bedbrook *et al.*, 2017; Yang *et al.*, 2018, 2019; Li *et al.*, 2019; Benes *et al.*, 2020; Bonetta and Valentino, 2020; Zhu *et al.*, 2020; Biswas *et al.*, 2021; Wittmann *et al.*, 2021; Brandes *et al.*, 2022; Hsu *et al.*, 2022). ML largely consists of ‘supervised’ tasks that involve training ML algorithms on previously seen protein sequences (e.g. enzyme sequence) with associated labels (e.g. catalytic activity). The trained model can then be used to predict labels of previously unseen but similar data inputs (Yang *et al.*, 2019; Mazurenko *et al.*, 2020; Newman and Furbank, 2021; Wittmann *et al.*, 2021). Several examples exist of ML applications being used to screen enzyme properties; however no model exists which has predicted Rubisco kinetics from sequence variation (Romero *et al.*, 2013; Yang *et al.*, 2018; Greenhalgh *et al.*, 2021; Hsu *et al.*, 2022). The reasons for this may be that we do not know exactly which properties of the Rubisco protein determine Rubisco kinetics. Additionally, state-of-the-art ML algorithms such as neural networks usually require hundreds or thousands of labelled data to perform well; that is not possible with the current size of Rubisco datasets.

Gaussian processes (GPs), a family of non-parametric, non-linear Bayesian models, have shown to predict enzyme properties such as thermostability and activity, given a limited amount of experimental data (Rasmussen and Williams, 2006; Yang *et al.*, 2018, 2019; Deringer *et al.*, 2021; Dutordoir *et al.*, 2021). A GP finds non-linear functions f(x1), f(x2)that map the relationship of similar labels (e.g. catalytic activity) with similar inputs x1,x2(e.g. enzyme sequences), as encoded by a kernel function (Jokinen *et al.*, 2018; Greenhalgh *et al.*, 2021). The kernel function measures the similarity of the input data in the form of a covariance matrix. A key feature of a GP is that it can characterise the model uncertainty due to lack of similar data, which can be used to determine the quality of predictions.

With all ML techniques, protein sequences must be transformed into numerical representations and performance can suffer if the protein sequences are not encoded correctly. It is difficult to suggest *a priori* the best way to numerically represent protein sequences, as they can be represented on a number of different levels, such as physiochemical properties of amino acids or the three-dimensional structure. Over the past decade, two classes of encoding schemes have been tested for mapping protein sequence-function relationships. A classical encoding scheme (or ‘one-hot encoding’) directly represents a sequence of amino acids in binary notation, and a ‘learned encoding’ scheme involves training an unsupervised ML method on millions of unlabelled protein sequences (Yang *et al.*, 2018; Alquraishi, 2021; Elnaggar *et al.*, 2021; Rives *et al.*, 2021; Wittmann *et al.*, 2021). After the learned encoding scheme has been trained it can be reused to produce numerical vector representations of protein sequences (Elabd *et al.*, 2020; Faulon and Faure, 2021; Wittmann *et al.*, 2021). The learned encoding scheme assumes that all protein sequences follow a set of evolutionary rules or biophysical traits that govern the relationships between protein sequences that allow them to carry out a biological function (Elabd *et al.*, 2020; Faulon and Faure, 2021; Wittmann *et al.*, 2021). The vector representations from the learned encoding scheme capture the relationships between proteins from the learned sequence-space. As result, similar sequences will have similar vector representations, and so can be assumed to have similar biological function by a downstream-supervised ML model such as a GP (Elabd *et al.*, 2020; Faulon and Faure, 2021; Wittmann *et al.*, 2021).

We think that the above ML processes could map the Rubisco sequence-function landscape for predicting unmeasured Rubisco kinetics. Previously, it was shown that Rubisco kinetic trade-offs exist between the Sc/o, Kcat and Michaelis-Menten constant for CO_2_ (Kc), leading to the belief that Rubisco kinetics are heavily constrained within a low-dimensional landscape (Tcherkez *et al.*, 2006; Savir *et al.*, 2010). However, recent work highlighted the importance of phylogenetic constraints for Rubisco kinetics, suggesting that closely related species are more likely to have similar kinetics (Flamholz *et al.*, 2019; Bouvier *et al.*, 2021, 2022); but see exceptions driven by a rapid evolution within recent adaptive radiations (Kapralov and Filatov, 2006; Kubien *et al.*, 2008; Kapralov *et al.*, 2011; Galmés *et al.*, 2014a) Thus, similarity of Rubisco sequences might be among the many features that GPs with protein encoding schemes may use for interpolating uncharacterized Rubisco kinetics.

Here, we trained GPs with either a learned encoding scheme or classical encoding scheme on form IB Rubisco sequences and kinetic data from C_3_ and C_4_ plant species. We evaluated the performance of the ML frameworks using leave-one-out cross validation, and found that the GPs with the learned encoding scheme outperformed the classical encoding scheme. Next, we subjected the GPs with the learned encoding scheme to another validation framework to detect overfitting. This involved removing species sharing the same genus during model training, and using the unseen genus group to assess model performance; from here on referred to as ‘leave-genus-out’ cross validation. We found that the GPs with a learned encoding scheme generalized across plant genera well. Finally, we wanted to validate hundreds of predictions without experimental data. One strategy of doing this was grouping predictions by photosynthesis type and taxonomical group for which mechanisms have been hypothesized to constrain Rubisco kinetics.

## Materials and methods

### Rubisco kinetics and sequence data

Rubisco large subunit harbouring the catalytic site is encoded by the *RbcL* gene, which therefore has a major influence on Rubisco kinetic properties (Kellogg and Juliano, 1997; Camel and Zolla, 2021). From the literature, 165 C_3_ and C_4_ plant Rubisco *in vitro* Kcat values (25 °C pH near 8), 170 *in vitro* Sc/o values, and 170 *in vitro* Kc values, as well as corresponding RbcL sequences, were obtained (Supplementary Table S1; Jordan and Ogren, 1983; Lehnherr *et al.*, 1985; Uemura *et al.*, 1997; Kubien *et al.*, 2008; Savir *et al.*, 2010; Viil *et al.*, 2012; Galmés *et al.*, 2014a, b; Hermida-Carrera *et al.*, 2016; Prins *et al.*, 2016; Sharwood *et al.*, 2016; Long *et al.*, 2018; Flamholz *et al.*, 2019). If studies reported overlapping *in vitro* kinetic data, the duplicate from the most recent study was kept and the other duplicate(s) discarded. Additional corrections were made to the data as follows: Standard errors (SE) with reported kinetic values such as Kcat, Kc, and Sc/o were converted to standard deviations (SD) using the number of species and/or replicates. When the number of replicates and/or species were not reported, the number of measurements were assumed to be from one sample. When the number of replicates and/or species were reported as a range (e.g. *n*=6–10) the mean number of samples was taken. Kc measurements under anoxygenic conditions were adjusted to ambient O_2_ conditions (Kc^21%O2^) using the following equation: $Kc^{21\;\%\;O2}=Kc^{O\;\%\;O2}\cdot (1+\frac{O_{2}}{K_{o}})$(Von Caemmerer, 2000), where ‘Kc^0%O2 ‘^ refers to Kc measured under anoxygenic conditions, ‘O_2_’ refers to the ambient O_2_ concentration and ‘Ko’ refers to the Rubisco Michaelis-Menten constant for O_2_ (μM).

### Model setup

**Fig. 1.**
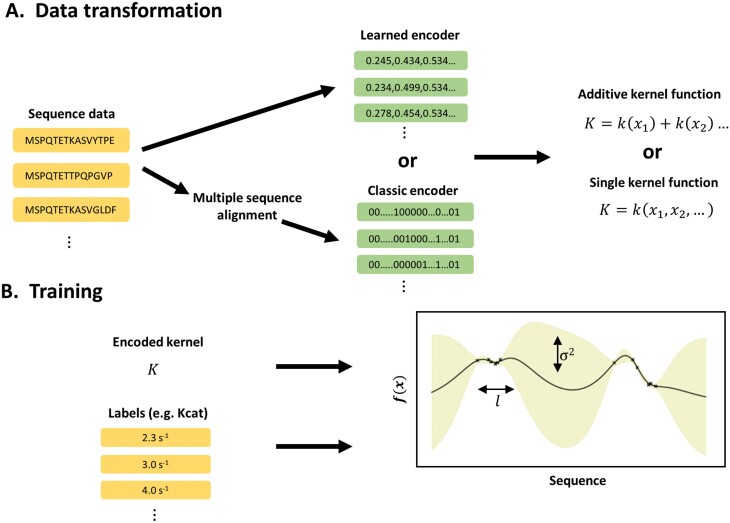
Schematic diagram showing steps involved in training a Gaussian process (GP) regression. (A) Rubisco large subunit (RbcL) sequences can be converted to either a binary representation (classical encodings) which explicitly represents the amino acids or learned encodings (such as Rives *et al.*, 2021) which involves another machine learning method - learning key features of each sequence (such as physiochemical properties or secondary structures) and storing these features as numerical vectors. The encoded RbcL sequences are stored in a kernel which describes the similarity between the encoded sequences. A kernel function can be applied to each input feature of the encodings. For example, $k(x_{1})$would encode the first numerical input for the learned encodings or the first alignment position for the classical encodings. Alternatively, input features can vary simultaneously using a single kernel function. (B) During model training, hyperparameters such as the length scale (l) and/or variance $\left(\sigma^{2}\right)$are optimised to find functions ( f(x)) that describe the relationship between the RbcL encodings and associated labels (e.g. turnover rate: Kcat). The ldescribes the horizontal distances betweenf(x), and $\sigma^{2}$ the vertical distance (i.e. noise and signal). As such, GPs provide a flexible framework for explaining numerous relationships.

A schematic diagram of the ML procedure is shown in Fig. 1. Just like a simple linear model, a GP can be used for regression or classification tasks (Rasmussen and Williams, 2006 ). Here, since kinetics are continuous variables, a GP regression was used. All ML tasks were performed using the Python ‘GPflow’ module (version 2.1; Matthews *et al.*, 2017) and packaged into user-friendly Google Colab notebooks (https://github.com/Iqbalwasim01/Mining-Rubisco-kinetics.git).

*Protein encoding scheme*. Two protein encoding schemes were tested before choosing a final encoding scheme. The classical encoding scheme (or one-hot encoding) expresses each amino acid as a 20 digit vector with the value ‘1’ indicating the identity and position of the current amino acid out of 20 other amino acid types, which are represented with the value ‘0’ (Yang *et al.*, 2018; Bonetta and Valentino, 2020; Elabd *et al.*, 2020). The one-hot encoding scheme is a relatively sparse and memory inefficient representation of protein sequences. For example, RbcL with a length of 450 amino acids would result in a 9000 length vector. Furthermore, ‘one-hot encoding’ requires that all RbcL sequences are aligned to the same length, and each time a new sequence is added the alignment procedure must be repeated. Here, an alignment procedure was performed using the ‘msa’ R package with the ‘Clustal omega’ alignment algorithm (Bodenhofer *et al.*, 2015).

However, the learned encoding scheme takes inspiration from natural language processing, and involves a semi-supervised ML model, learning basic underlying laws or rules of protein sequences that allow proteins to carry out a biological function (Yang *et al.*, 2018; Bonetta and Valentino, 2020; Elabd *et al.*, 2020; Wittmann *et al.*, 2021). The learned encoding scheme also known as ESM-1b based on a neural network with a transformer architecture (Rives *et al.* 2021) was adopted. Previous studies have shown that it predicts residue to residue contacts and secondary structure better than other transformers (Rao *et al.*, 2019; Elnaggar *et al.*, 2021). The learned encoding scheme summarised each RbcL sequence as a vector of length 1280. Once the RbcL sequences were converted to either the classical or learned encoding scheme, the encodings served as the direct inputs into the GP regression (Fig. 1).

*GP covariance structure.* A GP regression defines a distribution over functions linking data inputs (e.g. RbcL sequence encodings) with labels (e.g. kinetics). The functions are encoded by a kernel function represented as a covariance matrix and mean, which measure the similarity or nearness of input data (Rasmussen and Williams, 2006). The kernel function makes the basic assumption that data inputs (e.g. RbcL sequences), which are closely related are more likely to have similar labels, but some additional prior knowledge is required, such as whether the functions are linear, smooth, or rough. When the underlying nature is unknown, a popular choice of kernel is the non-linear ‘Matern 5/2’ kernel, which was used here (Rasmussen and Williams, 2006). A linear kernel function was also tested to demonstrate the need for the non-linear Matern 5/2 kernel. When data inputs consist of more than one numerical value, the kernel can be applied to each numerical value position allowing the GP regression to learn across multiple input positions known as an ‘additive kernel’ (Duvenaud *et al.*, 2011). For instance, many phenomena depend on the sum of parts; for example, the value of a car, which can be better approximated by the sum of prices of individual car parts. Similarly, the amino acid sites in a protein sequence may convey greater information when protein sequences share a high degree of overall structural similarity. Therefore, this study first applied the kernel function to each learned encoding input position or classical encoding alignment position i.e. $K=k\left(x_{1}\right)+k\left(x_{2}\right)\ldots$(Fig. 1). The performance with an additive kernel was then compared with a single kernel, where the GP depends on all input positions simultaneously i.e.$K=k\left(x_{1},x_{2},\ldots \right)$. The reason for testing both kernel configurations is that if the encodings consist of many low-order interactions, the additive kernel can exploit this and improve model performance (e.g. see Duvenaud *et al.*, 2011); if not, both the additive and single kernel configurations should give similar performance. Finally, during training, the kernel hyperparameters such as the length scale ‘$l^{\prime }$ and/or variance ${\;}^{\prime }\;{\sigma^{2}}^{\prime }$were tuned by maximizing the probability of observing the data points, known as the marginal likelihood. Predictions for new data inputs were then obtained from drawing samples from the trained GP.

### Leave-one-out cross validation

Performance of the GP regression was assessed using leave-one-out cross validation. Generally, any cross-validation involves splitting a dataset into training and testing datasets. The training dataset with input data (e.g. RbcL sequences) and labels (e.g. kinetics) is used to fit the GP regression model parameters, and the testing dataset with input data and labels is used to assess the performance of the trained GP regression to unseen data. Leave-one-out cross validation, as the name implies, involves holding out one labelled data input out of the training dataset and using the remainder of the dataset for fitting the GP model parameters, and predicting the unseen labelled data input that was left out. For example, if a dataset consists of 170 data inputs with labels, the model would be trained on 169 data inputs with labels, and the data input and label that was omitted would serve as the testing data set. Leave-one-out cross validation is carried out on each labelled data input, leaving a different labelled data input out of the training dataset each time. The predictions are gathered, and performance metrics such as coefficient of determination (R^2^) and mean absolute error (MAE) are calculated with the experimental data.

Leave-one-out cross validation was conducted for GP models with the learned and classical encoding schemes and different kernel configurations (i.e. single or additive and Matern 5/2 or linear).

### Leave-genus-out cross validation

The leave-one-out cross-validation aims to reduce the chance of model overfitting and assess model performance to unseen data. We know patterns or biases can arise from training models on similar datasets that could give a misleading picture of model performance. For instance, it is well known that form IB Rubiscos from the same genus can have similar sequences and kinetic properties (Hermida-Carrera *et al.*, 2016; Orr *et al.*, 2016). This could have led to overoptimistic performance metrics during leave-one-out cross validation, because at least one form IB variant from the same genus would have been left in the training dataset during model training. To see if the GPs generalize across genera, attempts were made to split the data equally, while ensuring that a genus group was left out of the training set each time. However, each genus group had unequal species numbers, which made it difficult to create equally distributed testing/training splits, while ensuring non-overlapping genus criteria. Instead, educated splits between the data were made by leaving a genus group out of the training data, and then testing the model on this omitted genus group. While the R^2^ metric was used in the leave-one-out cross validation for assessing performance, it is not suitable for assessing all areas of predictive performance, because it scales with the size of the dataset (i.e. the more data points there are, the less sensitive the R^2^ metric is to changes) and assumes values are strictly monotonically associated. Because each genus group contained unequal species numbers, were small, and predictions may not be normally distributed or monotonically associated with experimental values, model performance was assessed with the MAE metric as well as direct comparison with the experimental means ±SD.

### Benchmarking GP uncertainty estimates

A benefit of a GP is that a ${\;}^{\prime }\;{\sigma^{2}}^{\prime }\;$ estimate is provided with each prediction, which allows users to identify predictions with a high chance of being different from the training dataset. In other words, the lower the predicted $\sigma^{2}$, the nearer the prediction is to an example found in the training dataset. However, the GP $\sigma^{2}$ parameter is not explicitly dependent on the labels (i.e. kinetics), and is actually dependent on the data inputs (e.g. see Deringer *et al.* (2021). During training, the $\sigma^{2}$ parameter is implicitly mapped to the data labels via hyperparameter optimization. Because the $\sigma^{2}$ parameter is a trainable part of the model, the reliability of the $\sigma^{2}$ estimates must be assessed against test data. Here, the quality of the predicted $\sigma^{2}$ estimates from cross validation was first assessed using the Spearman rank correlation with the true errors (i.e. absolute errors between actual mean values and predicted mean values; Greenman *et al.*, 2022). Secondly, we assessed if the actual mean values fall within the 95% predicted confidence intervals (CIs; ±2σ), as demonstrated by Kompa *et al.* (2021). This method involves two metrics: ‘coverage’, which is if the actual mean value falls within the predicted 95% CI; and ‘width’, which is the full range of the predicted 95% confidence interval (4σ).

### t-distributed stochastic neighbour embedding (t-SNE)

In this study, protein encoding schemes convert protein sequences from their widely used amino acid format to sequences of numbers, which cannot be understood using conventional protein sequence analysis methods, such as multiple sequence alignments. To investigate how protein encoding schemes portray proteins, which ultimately determine their fate for functional prediction tasks, a dimensionality reduction method called t-distributed stochastic neighbour embedding (t-SNE) was applied (Maaten and Hinton, 2008). t-SNE projects the protein encodings into two-dimensions, which allows patterns/clustering arising from the protein encodings to be visualized. t-SNE was performed on the RbcL classical and learned encodings with a perplexity of 20 and default learning rate parameters using the ‘sci-kit learn’ Python module (version 1.0.2; Pedregosa *et al.*, 2011).

### K-nearest neighbour (KNN)

K-nearest neighbour (KNN) with the Levenshtein distance, a simple unweighted global alignment distance, has shown to predict enzyme activity from sequence data (Biswas *et al.*, 2021; Bryant *et al.*, 2021). KNN has been adopted in recent studies as a simple baseline method (Goldman *et al.*, 2022). KNN models were built on the same tasks as the GP models using the ‘sci-kit learn’ Python (version 1.0.2) and ‘editdistance’ (version 0.3.1) modules. KNN ‘number of neighbours’ was treated as a hyperparameter and chosen during leave-one-out cross validation.

### Assessing RbcL sequence-space predictions with trait data

Wild type RbcL sequences from non-redundant protein databases were obtained (*n*=35 413) from a recent search (Davidi *et al.*, 2020). Unknown species, sequences with lengths >500 or <450 and duplicate entries were omitted, leaving 13 124 unique RbcL sequences. From these, 9052 RbcL sequences identified as land plants (Embryophyta) remained. Using the fully trained GPs with the chosen encoding scheme, Rubisco kinetic predictions were obtained for 9052 land plants. Predictions were grouped by plant photosynthetic type (C_3_, C_4_, or crassulacean acid metabolism (CAM) and taxonomical group (Angiosperms, Bryophytes, Gymnosperms, and ‘Ferns’; the latter is a group that included Pteridophyta and Lycopodiophyta). Differences between groups were assessed using one-way ANOVA and Duncan’s post hoc test with the ‘DescTools’ R package (version 0.99.44).

While the sequence criteria of <500 and >450 was used to remove incomplete sequences, some sequences may still have several amino acids missing from the N-terminus and/or C-terminus, or ambiguous amino acids, which could have led to high predicted$\sigma^{2}$. To see if such sequences affected the distribution of predictions, predictions were restricted based on $\sigma^{2}$ estimates selected from cross validation, if the $\sigma^{2}$ estimates were well calibrated. Otherwise, the influence of outliers was assessed by removing predictions outside the training dataset ranges. Predictions were grouped by plant photosynthetic type and taxonomical group, as described above.

## Results

### GP performance with a learned encoding scheme compared with a classical encoding scheme

**Fig. 2.**
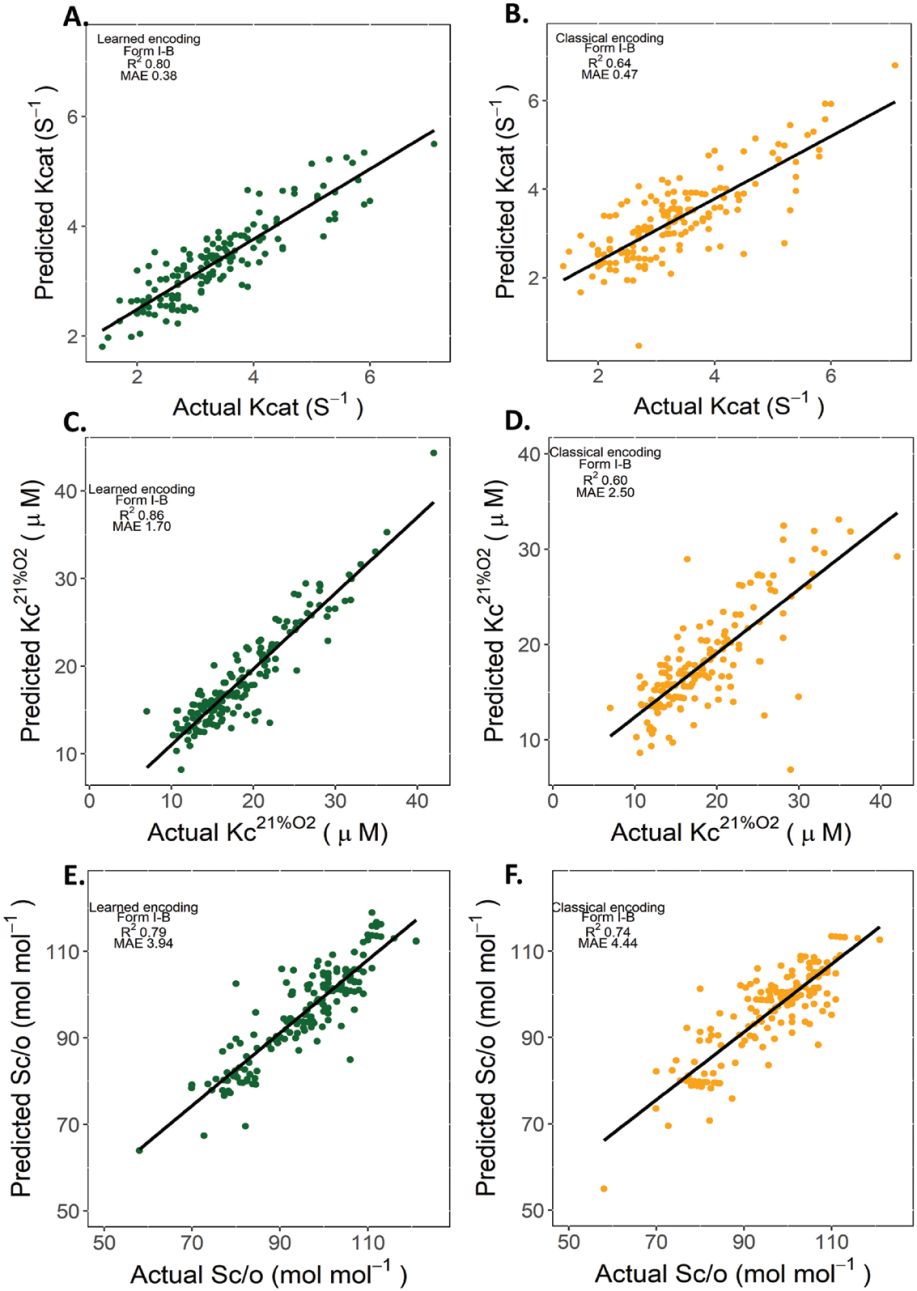
Comparison between predicted and actual carboxylation turnover rate (Kcat: s^-1^), Michaelis-Menten constant for CO_2_ at ambient O_2_ (Kc^21%O2^: µM) and specificity for CO_2_ over O_2_ (Sc/o: mol mol^-1^) at 25 °C. The performance was determined using leave-one-out cross-validation with the learned encoding scheme (Rives *et al.*, 2021) (green) and classical encoding scheme (orange). The better performance of the learned encodings with an additive non-linear kernel justified the adoption of this method over classical for the final machine learning tasks.

GPs with the learned encoding and classical encoding schemes were trained on form IB RbcL sequence and kinetic data. The performance of the two encoding schemes applied to a single and additive kernel configuration was assessed (Supplementary Figs S1-S3). The GPs with the learned encodings applied to an additive non-linear Matern 5/2 kernel had the highest predictive ability (Fig. 2; R^2^ 0.79–0.86) compared with the classical encodings (R^2^ 0.60–0.74) and other kernel configurations (Supplementary Figs S1-S3). When the neural network of the learned encoding scheme was randomly initialized with untrained weights, the boost in performance remained (Supplementary Table S2). Therefore, this suggested that the learned encoding scheme was most likely driven by the neural network architecture rather than the pre-trained weights. The KNN (baseline) models had similar performance to the GPs adopting a single kernel configuration or performed worse (i.e. Kcat; Supplementary Fig. S4). These results justified the adoption of the learned encodings with the non-linear Matern 5/2 additive kernel for the final models (Fig. 2).

### GP performance with the learned encoding scheme for numerous plant genera

Form IB Rubisco variants included as part of the training data could have led to overoptimistic performance metrics shown in Fig. 2, because at least one form IB Rubisco from the same genus may have been left in the training dataset during model training. Here, the GPs with the learned encoding scheme were assessed using another validation framework. This time form IB Rubiscos sharing the same genus were omitted from the model during training. The remaining data was used to train the model, and the omitted genus group was used to assess the model performance.

The GPs with the learned encoding scheme displayed excellent performance. The majority of genus groups had Kcat predictions with a MAE < 0.5 s^-1^ (Supplementary Fig. S5), Kc^21%O2^ predictions with a MAE < 4.00 μM (Supplementary Fig. S6) and Sc/o predictions with a MAE < 7.00 mol mol^-1^ (Supplementary Fig. S7).

### Visualization of the RbcL learned and classical encodings used during GP training

**Fig. 3.**
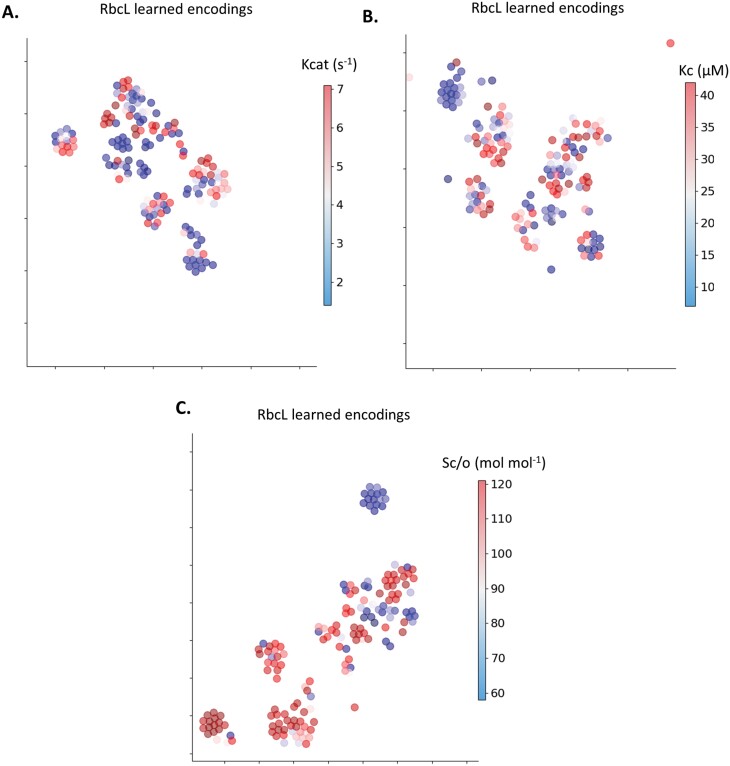
Visualization of the Rubisco large subunit (RbcL) learned encodings used in the fully trained Gaussian process (GP) models. Each data point represents an RbcL learned encoding with (A) carboxylation turnover rate (Kcat: s^-1^) (*n*=165); (B) Michaelis-Menten constant for CO_2_ at ambient atmospheric O_2_ (Kc^21%O2^: μM) (*n*=170); and (C) specificity for CO_2_ over O_2_ (Sc/o: mol mol^-1^) (*n*=170).

To investigate how the GPs learned to predict form IB Rubisco kinetics, the RbcL sequence classical and learned encodings used for model training were visualized using t-distributed stochastic neighbour embedding (t-SNE; Fig. 3; Supplementary Fig. S8). Both the classical and learned encodings showed some sequences with higher Kcat, Kc^21%O2^, and Sc/o clustered together, and some sequences with lower Kcat, Kc^21%O2^, and Sc/o clustered together. Differences between the RbcL classical and learned encodings were unclear for Sc/o, but more clustering in the learned encodings than the classical encodings could be seen for Kcat and Kc^21%O2^.

### Assessing GP uncertainty estimates

Generally, it is assumed that GP predictions with high $\sigma^{2}$ most likely arise from parts of the trained GP from which less similar training data was included. However, because the $\sigma^{2}$ estimates are a trainable part of the model, the reliability of the predicted $\sigma^{2}$ was assessed before guiding the selection of appropriate predictions.

The correlations between predicted $\sigma^{2}$ and true error from leave-one-out and leave-genus-out cross validation are shown in Supplementary Figs S9, S10. No clear trend was observed between predicted $\sigma^{2}$ and true error. The uncertainty from leave-genus-out cross validation assessed using coverage and width is shown in Supplementary Fig. S11. Most genus groups exhibited high coverage and varying average width (4σ) but some did not. As predicted mean values become increasingly out of distribution, ideal models should increase width, indicating model uncertainty while coverage remains high.

### Assessing RbcL sequence-space predictions with trait data

The final goal was to screen the kinetic properties of thousands of Rubisco variants *in silico* using the GPs with the learned encoding scheme. Predictions were made for 9052 unique RbcL sequences encoding Rubisco proteins from land plants. Grouping predictions by photosynthesis metabolism type revealed significant differences (*P*<0.05) between Kcat, Sc/o and Kc^21%O2^ of C_3_, C_4_, and CAM groups (Supplementary Fig. S12). Grouping predictions by taxonomical group revealed significant differences (*P*<0.01) between most groups, except the Kcat of angiosperms and ferns, and Kc^21%O2^ of gymnosperms and bryophytes (Supplementary Fig. S13).

**Fig. 4.**
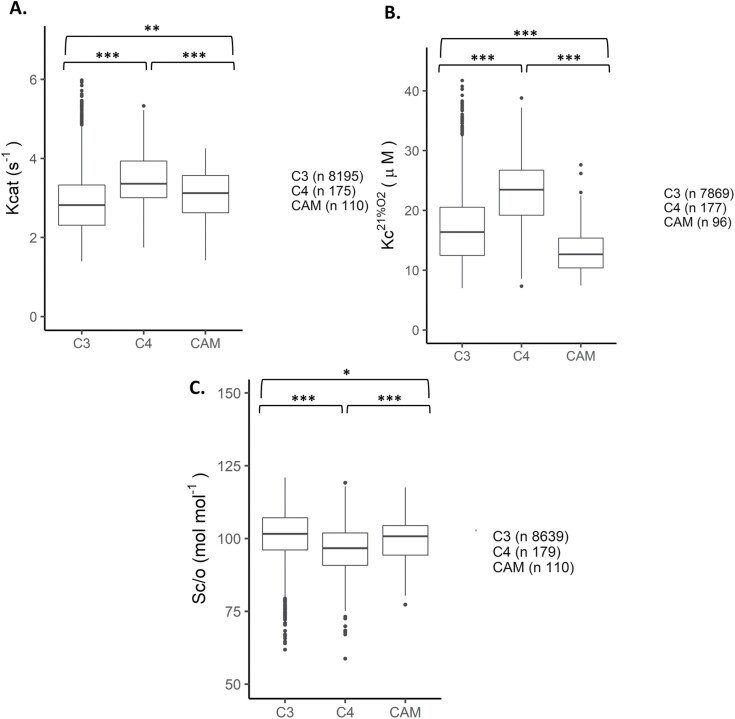
Predictions of land plant Rubiscos from different photosynthetic groups. Box plots depict (A) carboxylation turnover rate (Kcat: s^-1^); (B) Michaelis-Menten constant for CO_2_ at ambient atmospheric O_2_ (Kc^21%O2^: μM); and (C) specificity for CO_2_ over O_2_ (Sc/o: mol mol^-1^) predictions made using the fully trained Gaussian process (GP) models with the learned encoding scheme. Data shown are predictions within the ranges of the training dataset for Kcat (1.4, 7.1), Kc^21%O2^ (7, 42) and Sc/o (58, 121). Predictions were grouped by photosynthesis metabolism type (C_3,_ C_4_, or CAM). Box plot horizontal lines show the median value, and the box and whisker represent the 25^th^ and 75^th^ percentile and minimum to maximum distributions of the data. Significant differences from the one-way ANOVA with Duncan’s post-hoc test are shown for groups: ***P < 0.001, **P < 0.01, *P < 0.05; n.s., non-significant.

**Fig. 5.**
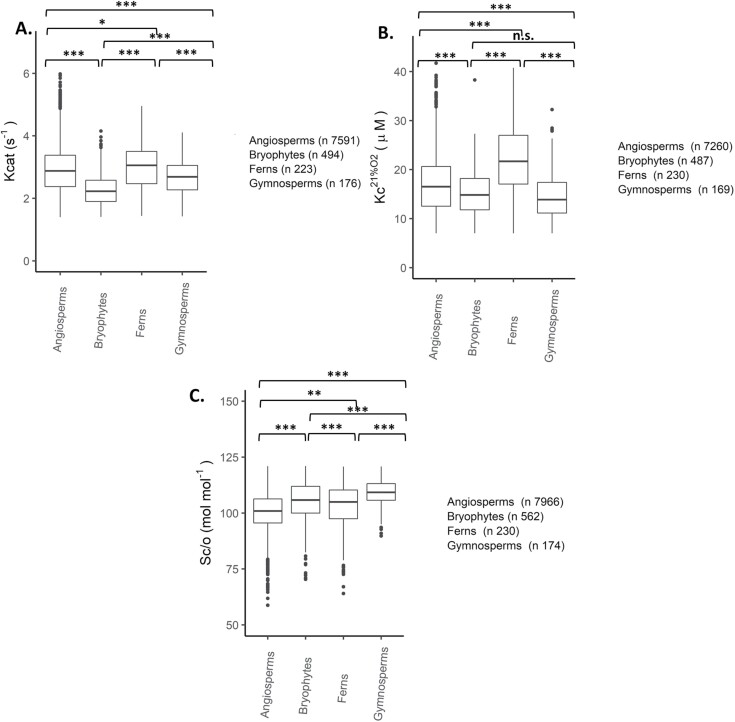
Predictions of land plant Rubiscos from different taxonomical groups. Box plots depict (A) carboxylation turnover rate (Kcat: s^-1^); (B) Michaelis-Menten constant for CO_2_ at ambient atmospheric O_2_ (Kc^21%O2^: μM); and (C) specificity for CO_2_ over O_2_ (Sc/o: mol mol^-1^) predictions made using the fully trained Gaussian process (GP) models with the learned encoding scheme. Data shown are predictions within the ranges of the training dataset for Kcat (1.4, 7.1), Kc^21%O2^ (7, 42) and Sc/o (58, 121). Predictions were grouped by taxonomical type (Angiosperms, ‘Ferns’ (including Pteridophytes and Lycopodiophytes), Gymnosperms or Bryophytes). Box plot horizontal lines show the median value, and the box and whisker represent the 25^th^ and 75^th^ percentile and minimum to maximum distributions of the data. Significant differences from the one-way ANOVA with Duncan’s post-hoc test are shown for groups: ***P < 0.001, **P < 0.01, *P < 0.05; n.s., non-significant.

Because the predicted $\sigma^{2}$ estimates from cross validation showed no clear trend (Supplementary Figs S9-S11), a criteria for determining the quality of predictions in the absence of experimental data could not be specified. Instead, the influence of outliers was assessed by removing predictions outside the ranges of the training dataset. Most kinetic predictions for Kcat (1.4, 7.1), Kc^21%O2^ (7, 42), and Sc/o (58, 121) were within the range (Fig. 4 versus Supplementary Fig. S12; Fig. 5 versus Supplementary Fig. S13). The overall trend in kinetics remained the same as before. For instance, Rubisco enzymes from CAM and C_4_ plants have a higher median Kcat than Rubisco enzymes from C_3_ plants. Similarly, the overall trend remained the same when grouping predictions by taxonomical type. For instance, angiosperms and ferns have a higher median Kcat than bryophytes and gymnosperms.

## Discussion

This work presents a useful tool for screening and predicting plant Rubisco kinetics for engineering efforts, as well as for fundamental studies on Rubisco evolution and adaptation. Advancements in protein language modelling has allowed the exploitation of existing plant Rubisco data for predicting Rubisco kinetics *in silico*. Furthermore, our predictions followed well established trends observed by previous studies in plants with different photosynthetic types without *a priori* knowledge. For example, generally Rubisco proteins from C_4_ plants have a higher Kcat, Kc^21%O2^ and lower Sc/o than those from C_3_ plants (Galmés *et al.*, 2014c, 2015, 2019; Hermida-Carrera *et al.*, 2016; Prins *et al.*, 2016; Iñiguez *et al.*, 2020). In contrast, CAM plants have a mean Kcat similar to that of C_4_ plants (Hermida-Carrera *et al.*, 2020; Iñiguez *et al.*, 2020).

The kinetic properties of modern Rubisco proteins are believed to be shaped by changes in atmospheric CO_2_ and O_2_ concentrations, and temperature over time (Tcherkez *et al.*, 2006, 2018; Savir *et al.*, 2010; Studer *et al.*, 2014; Hermida-Carrera *et al.*, 2016; Cummins *et al.*, 2018; Moore *et al.*, 2021). C_4_ and CAM plants both possess carbon concentrating mechanisms (CCMs) that enhance CO_2_ concentration near the Rubisco active site (Raven and Beardall, 2014; Raven *et al.*, 2017; Young and Hopkinson, 2017; Ruban *et al.*, 2022). CCMs in C_4_ and CAM plants may have first arisen in environments with a high O_2_:CO_2_ ratio, and a decrease in O_2_:CO_2_ ratio over several million years led to the present day maintenance of high Kcat values to cope with higher mesophyll CO_2_ concentrations (Cc; Iñiguez *et al.*, 2020). Because both C_4_ and CAM plants are also found in high temperature environments, CCMs also help concentrate CO_2_ near the active site of Rubisco when the gas solubility of atmospheric CO_2_:O_2_ ratio decreases with increasing temperature (Raven *et al.*, 2017; Iñiguez *et al.*, 2020). Despite the presence of CCMs in both C_4_ and CAM plants and similar mean Kcat values, both groups had significantly different mean Kc^21%O2^ and Sc/o. C_4_ plants may have evolved higher Kc^21%O2^ and lower Sc/o because adoption of the CCMs led to a reduced requirement for a higher Sc/o and lower Kc^21%O2^ (Iñiguez *et al.*, 2020). On the other hand, unlike C_3_ and C_4_ plants, CAM plants have evolved to fix CO_2_ over the course of a day in phases, and are commonly found in drier climates (Leverett *et al.*, 2021; Ruban *et al.*, 2022). One possibility is that the temporal separation of CAM CO_2_ fixation may hinder the use of CCMs during some periods, leading to the requirement for a similar mean Sc/o to that of C_3_ plants, and lower mean Kc^21%O2^ (Iñiguez *et al.*, 2020).

Additionally, land plant Rubisco proteins are characteristic of the ecological or taxonomical group from which they originated (Fig. 5; Galmés *et al.*, 2014c). For instance, angiosperms have the largest distribution in kinetics because it is the largest and most diverse group of land plants comprising Rubisco proteins from C_3_, C_4_, and CAM plants.

What is unclear is how the GPs mapped the Rubisco sequence-function landscape. Projecting the classical and learned encodings suggests that some encodings with similar kinetics cluster together, but some do not (Fig. 3; Supplementary Fig. S8). Instead, the GPs may have found something ‘deeper’ about the relationship between RbcL encodings and kinetics during the training process. During training, when a single kernel function was applied over all encoding input positions, the models performed poorly compared with an additive kernel. This suggests a complex relationship which depends on the sum of small functions, rather than on a single large modelled function. Furthermore, GP models adopting a non-linear additive kernel and learned encodings had greater performance than the classical encodings (Fig. 2) and KNN baseline models (Supplementary Fig. S4). This reaffirms that learned representations of protein sequences improves performance of protein sequence-function tasks when some features of the relationship are unknown (Yang *et al.*, 2018; Rives *et al.*, 2021; Goldman *et al.*, 2022).

There are several strengths and limitations of the techniques used in this study. Firstly, one can assume that the training dataset only represented a fraction of all land plant Rubisco diversity. As a starting point, the first logical step was to test the models on this currently available data, before spending more time and resources on creating a more comprehensively rich training dataset that may reveal more subtle parts of the sequence-function landscape (Hsu *et al.*, 2022). In fact, when removing predictions outside the ranges of the training dataset (e.g. Fig. 4 versus Supplementary Fig. S12), there was no change in the kinetic trends, suggesting that predictions for most land plant Rubisco proteins are similar to the training dataset. We would be cautious about extending the current trained models to other Rubisco forms such as those found in bacteria and archaea, which exhibit greater sequence and kinetic diversity than form IB Rubisco proteins. For example, Davidi *et al.* (2020) identified form II Rubisco proteins with the fastest having a Kcat of 22 s^-1^, which is far greater than all known plant Rubisco proteins. As more experimental data becomes available, we expect models on more Rubisco forms to be built. 

Secondly, the models in this study assumed that features of RbcL determine the kinetic properties of form IB Rubisco proteins. Over the past few years this assumption is largely thought to be true because (i) the active site is encoded by the RbcL sequence, and (ii) the RbcL sequence is largely conserved over time as chloroplast-encoded genes evolved slower than nuclear-encoded genes (Kelly, 2021). It is now well established that the Rubisco small subunit encoded by the *RbcS* gene can influence catalysis too (Spreitzer *et al.*, 2005; Genkov and Spreitzer, 2009; Atkinson *et al.*, 2017; Martin-Avila *et al.*, 2020; Lin *et al.*, 2021; Sakoda *et al.*, 2021; Mao *et al.*, 2022). It would be interesting to see if incorporating RbcS sequences alongside RbcL sequences could improve the predictive power of our models. However, incorporating the RbcS *in silico* is further complicated by the existence of multiple *RbcS* genes located in the nucleus, and different nuclear-encoded *RbcS* genes differentially influencing Rubisco kinetics in the same plant (Khumsupan *et al.*, 2020; Martin-Avila *et al.*, 2020). Furthermore, the models in this paper can be used in experiments to predict the kinetics of novel Rubisco variants created *in silico* by manipulation of the Rubisco sequence, potentially creating better enzymes. Lastly, one benefit of using GPs is that predicted σ^2^ estimates are provided with predicted means, which allows users to identify predictions with a high chance of being different from the training dataset. Alternatively, one could assume that the higher the σ^2^ estimate, the greater the uncertainty in the predicted mean. The quality of the predicted σ^2^ estimates was judged (Supplementary Figs S9-S11), and the predicted means appear well calibrated against experimental data (Fig. 2) but the predicted σ^2^ estimates are not. One possibility is that the predicted σ^2^ estimates exhibit what is known as ‘sharpness’ because of the highly similar nature of the training dataset; the idea of ‘sharpness’ is that most predictions have small σ^2^ estimates, and larger σ^2^ are likely to appear once predictions are made for sequences outside the bounds of the training dataset (Tran *et al.*, 2020). In future work, we aim to collect more experimental data for model training which will allow a wider evaluation of the predicted σ^2^ estimates.

Overall, this study is the first to demonstrate the prediction of land plant Rubisco kinetics from RbcL sequence data. This study provides plant biologists with a pre-screening tool for highlighting Rubisco species exhibiting better kinetics for crop engineering efforts. Going forward, we expect more experimental data to become available, which will facilitate the development of richer models.

## Supplementary data

The following supplementary data are available at *JXB* online. 

Fig. S1. Leave-one-out cross validation results for GPs using a single Matern 5/2 kernel.

Fig. S2. Leave-one-out cross validation results for GPs using an additive linear kernel.

Fig. S3. Leave-one-out cross validation results for GPs using a single linear kernel.

Fig. S4. Leave-one-out cross validation results for KNN (baseline) models.

Fig. S5. Leave-genus-out cross validation plots for Kcat.

Fig. S6. Leave-genus-out cross validation plots for Kc^21%O2^.

Fig. S7. Leave-genus-out cross validation plots for Sc/o.

Fig. S8. Visualization of the RbcL classical encodings used during GP training.

Fig. S9. Spearman rank correlations of the leave-one-out cross validation predicted uncertainties and true errors.

Fig. S10. Spearman rank correlations of the leave-genus-out cross validation predicted uncertainties and true errors.

Fig. S11. Leave-genus-out cross validation predicted uncertainties assessed using the coverage and width method.

Fig. S12. Box plots depicting kinetic predictions for all land plant Rubisco proteins grouped by photosynthesis type.

Fig. S13. Box plots depicting kinetic predictions for all land plant Rubisco proteins grouped by taxonomical type.

Table S1. Rubisco experimental kinetics and Rubisco large subunit (RbcL) sequences for training Gaussian process models.

Table S2. Average performance of the learned encoding scheme across five randomly chosen sets of untrained weights.

erac368_suppl_Supplementary_Figures_S1-S13_Tables_S1-S2

## Data Availability

The data that support the findings of this study including kinetic predictions, sequence data and Google COLAB notebooks are openly available at GitHub (https://github.com/Iqbalwasim01/Mining-Rubisco-kinetics.git).

## Abbreviations

CAM: Crassulacean acid metabolism;
CCM: Carbon concentrating mechanism
GP: Gaussian process
Kc: Michaelis-Menten constant for CO_2_
Kc^21%O2^: Michaelis-Menten constant for CO_2_ at ambient O_2_
Kcat: Carboxylation turnover rate
*l*: Length scale
MAE: Mean absolute error
RbcL: Rubisco large subunit
RbcS: Rubisco small subunit
Rubisco: Ribulose-1,5-bisphosphate carboxylase/oxygenase
Sc/o: Specificity for CO_2_ over O_2_
σ^2^: Variance
t-SNE: t-distributed stochastic nearest neighbour
