# Supplementary material for: Predicting plant Rubisco kinetics from RbcL sequence data using machine learning
Source: J Exp Bot. 2022 Sep 12;74(2):638–50. doi: 10.1093/jxb/erac368 (PMC9833099; doi:10.1093/jxb/erac368)
Supplement: erac368_suppl_Supplementary_Figures_S1-S13_Tables_S1-S2 [file erac368_suppl_supplementary_figures_s1-s13_tables_s1-s2.pdf]

Supplementary material

Predicting plant Rubisco kinetics from RbcL sequence data using machine learning

[1]Wasim A Iqbal, [2]Alexei Lisitsa and [1]Maxim V Kapralov

[1] School of Natural and Environmental Sciences, Newcastle University, Newcastle upon Tyne, NE1 7RU, United Kingdom

[2] Department of Computer Science, University of Liverpool, Liverpool, L69 3BX, United Kingdom

Email: Maxim.Kapralov@ncl.ac.uk

Email : W.Iqbal@ncl.ac.uk

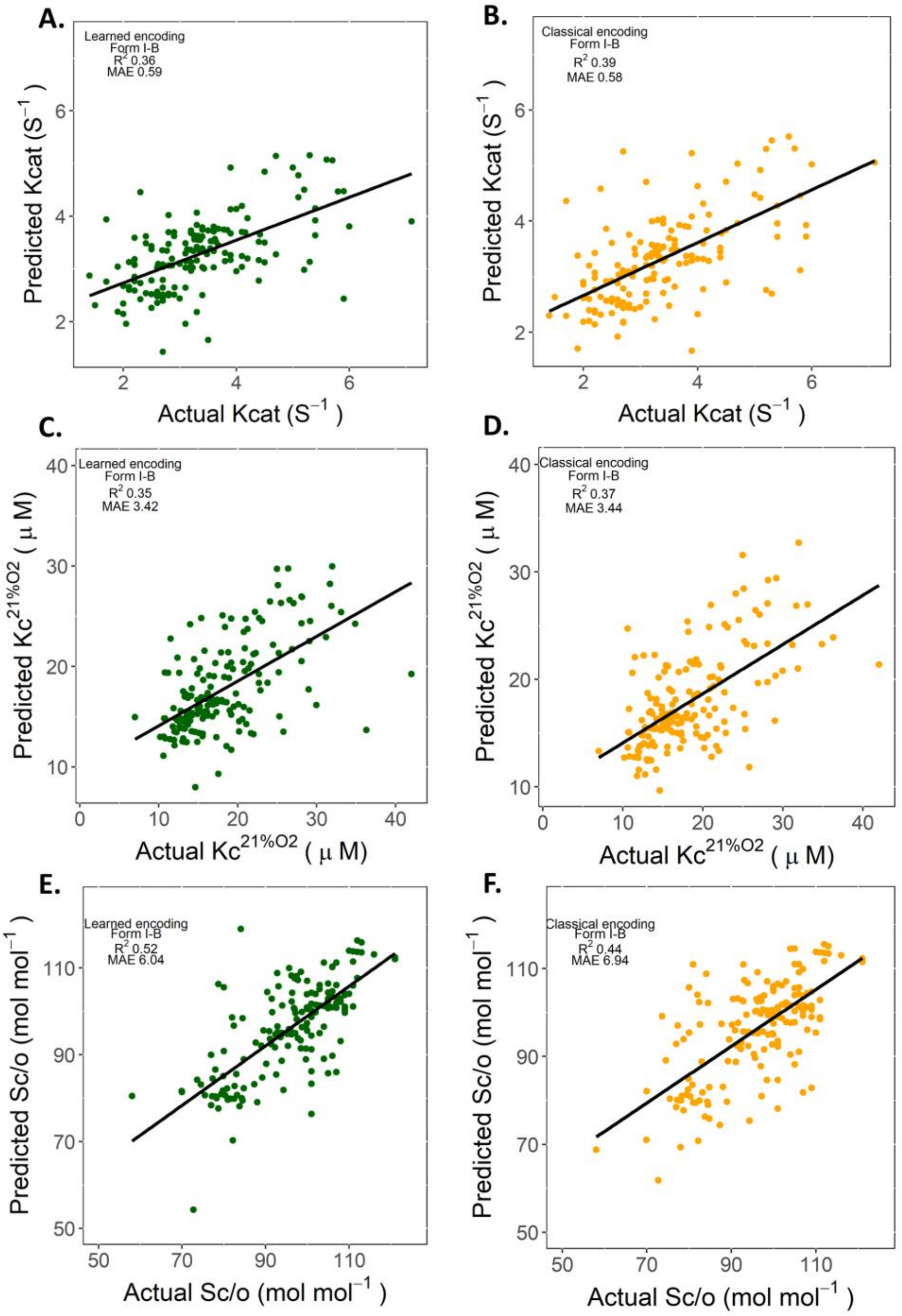

Figure S1. Comparison between predicted and actual carboxylation turnover rate (Kcat : s^{-1}), Michaelis-Menten constant for CO_2 at ambient O_2 ($Kc^{21\%O2}$: μM) and specificity for CO_2 over O_2 (Sc/o: mol mol^{-1}) at 25°C. Determined using leave-one-out cross-validation with the learned encodings (Rives et al., 2021) (green) and classical encodings (orange) encoded by a single Matern 5/2 kernel. The poor performance of the single Matern 5/2 kernel approach justified the use of an additive Matern 5/2 kernel for the final gaussian process models (see paper Figure 2).

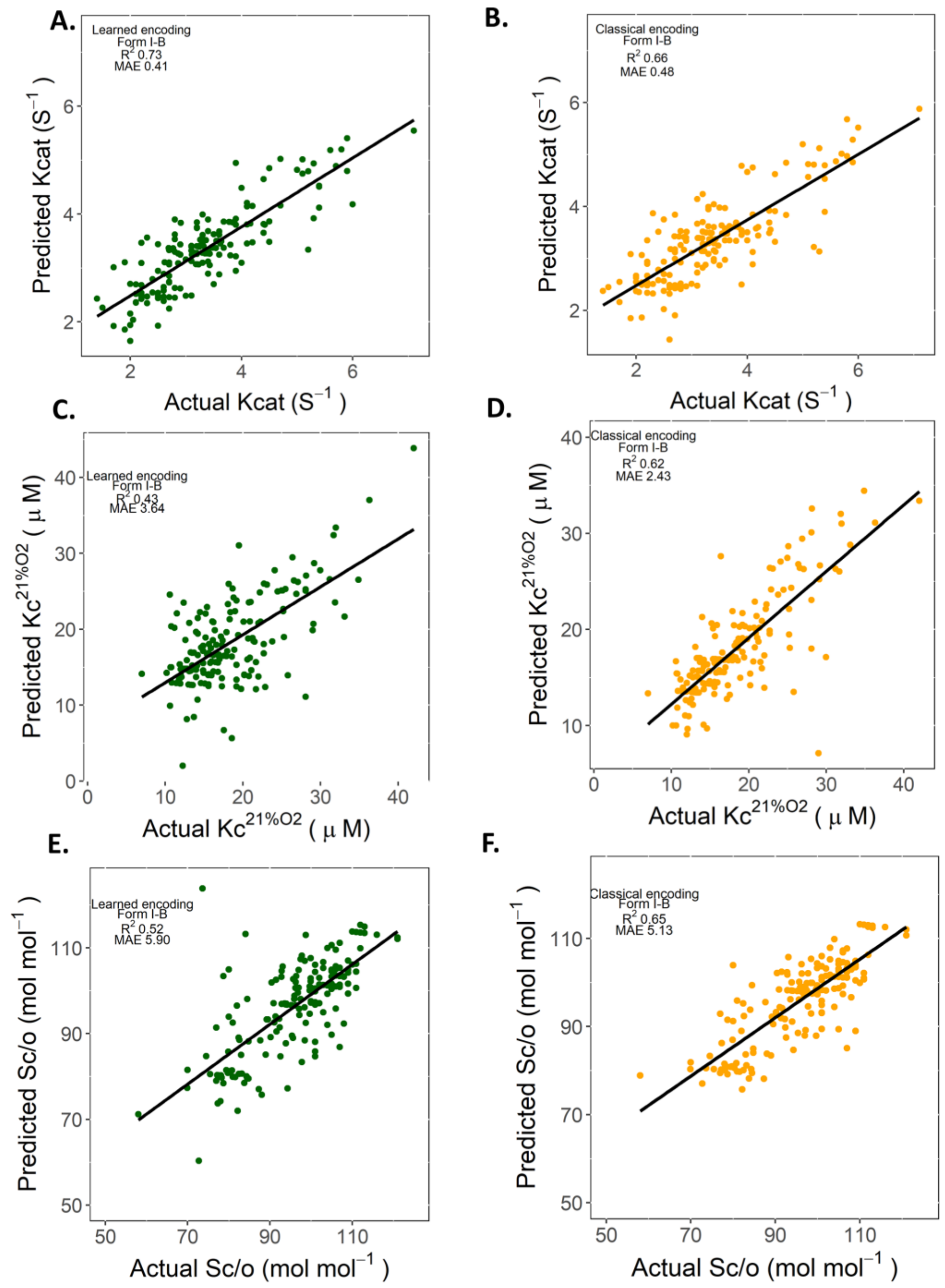

Figure S2. Comparison between predicted and actual carboxylation turnover rate (Kcat : s^{-1}), Michaelis-Menten constant for CO_2 at ambient O_2 ($Kc^{21\%O2}$: μM) and specificity for CO_2 over O_2 (Sc/o: mol mol^{-1}) at 25°C. Determined using leave-one-out cross-validation with the learned encodings (Rives et al., 2021) (green) and classical encodings (orange) encoded by an additive linear kernel. The poor performance of the additive linear kernel approach justified the use of an additive Matern 5/2 kernel for the final gaussian process models (see paper Figure 2).

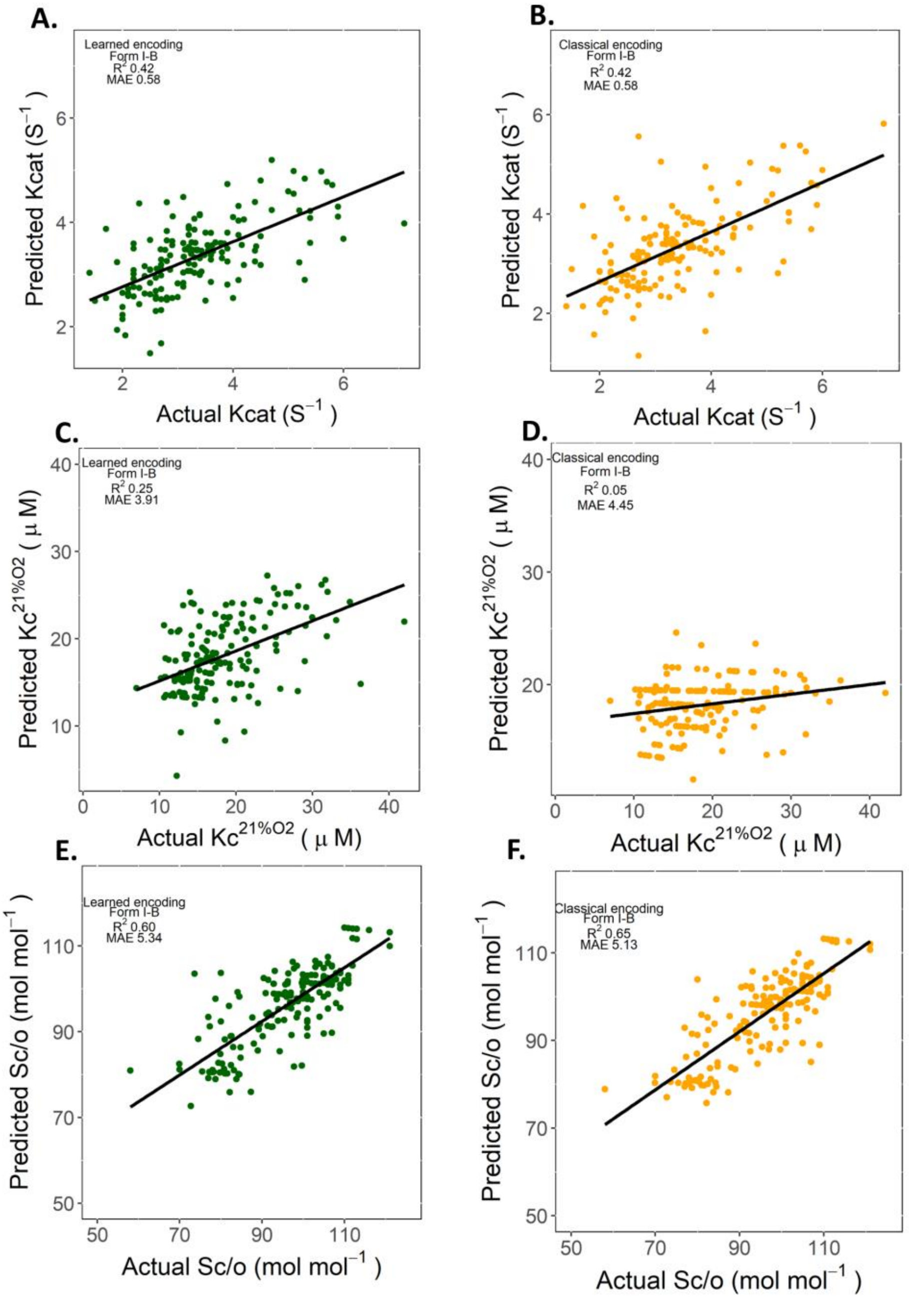

Figure S3. Comparison between predicted and actual carboxylation turnover rate (Kcat : s^{-1}), Michaelis-Menten constant for CO_2 at ambient O_2 ($Kc^{21\%O2}$: μM) and specificity for CO_2 over O_2 (Sc/o: mol mol^{-1}) at 25°C. Determined using leave-one-out cross-validation with the learned encodings (Rives et al., 2021) (green) and classical encodings (orange) encoded by a single linear kernel. The poor performance of the single linear kernel approach justified the use of an additive Matern 5/2 kernel for the final gaussian process models (see paper Figure 2).

Table S1. Average performance of the learned encoding scheme across five randomly chosen sets of untrained weights determined using leave-one-out cross validation.

Model configuration	Task	R^2	MAE
Additive Matern 5/2 kernel	Kcat	0.79 ± 0.01	0.40 ± 0.01
Additive Matern 5/2 kernel	$Kc^{21\%O2}$	0.82 ± 0.03	1.97 ± 0.17
Additive Matern 5/2 kernel	Sc/o	0.79 ± 0.01	4.03 ± 0.12
Single Matern 5/2 kernel	Kcat	0.30 ± 0.02	0.65 ± 0.01
Single Matern 5/2 kernel	$Kc^{21\%O2}$	0.25 ± 0.01	3.63 ± 0.04
Single Matern 5/2 kernel	Sc/o	0.66 ± 0.01	5.18 ± 0.06
Additive linear kernel	Kcat	0.63 ± 0.03	0.47 ± 0.02
Additive linear kernel	$Kc^{21\%O2}$	0.56 ± 0.06	3.16 ± 0.22
Additive linear kernel	Sc/o	0.70 ± 0.04	4.89 ± 0.32
Single linear kernel	Kcat	0.37 ± 0.02	0.60 ± 0.01
Single linear kernel	$Kc^{21\%O2}$	0.20 ± 0.03	3.94 ± 0.09
Single linear kernel	Sc/o	0.63 ± 0.01	5.41 ± 0.07

The learned encoding scheme is based on a fixed pre-trained neural network. Randomly assigning untrained weights led to minor differences in performance. This demonstrates that the boost in performance from the pretrained learned encoding scheme (e.g. Figure 2) is most likely driven by the neural network architecture. Mean ± SD performance metrics across five randomly chosen sets of untrained weights. Mean absolute errors (MAE) are in the units of the corresponding task outcomes i.e. Kcat (s^{-1}), $Kc^{21\%O2}$ (μM) or Sc/o (mol mol^{-1}).

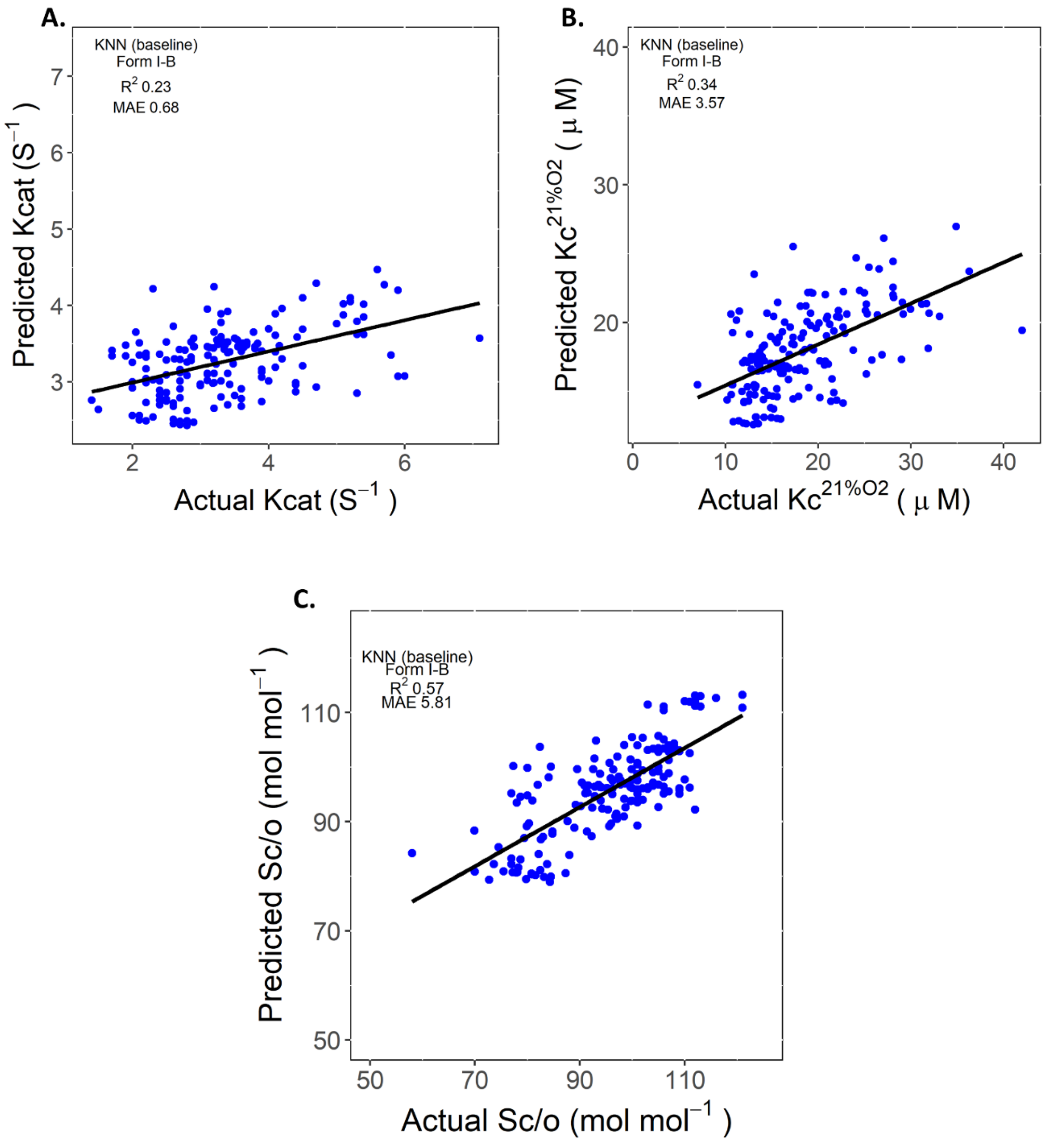

Figure S4. Comparison between predicted and actual carboxylation turnover rate (Kcat : s^{-1}), Michaelis-Menten constant for CO_2 at ambient O_2 ($Kc^{21\%O2}$: μM) and specificity for CO_2 over O_2 (Sc/o: mol mol^{-1}) at 25°C. Determined using leave-one-out cross-validation with K-nearest neighbour (KNN) sequence-similarity (baseline) models.

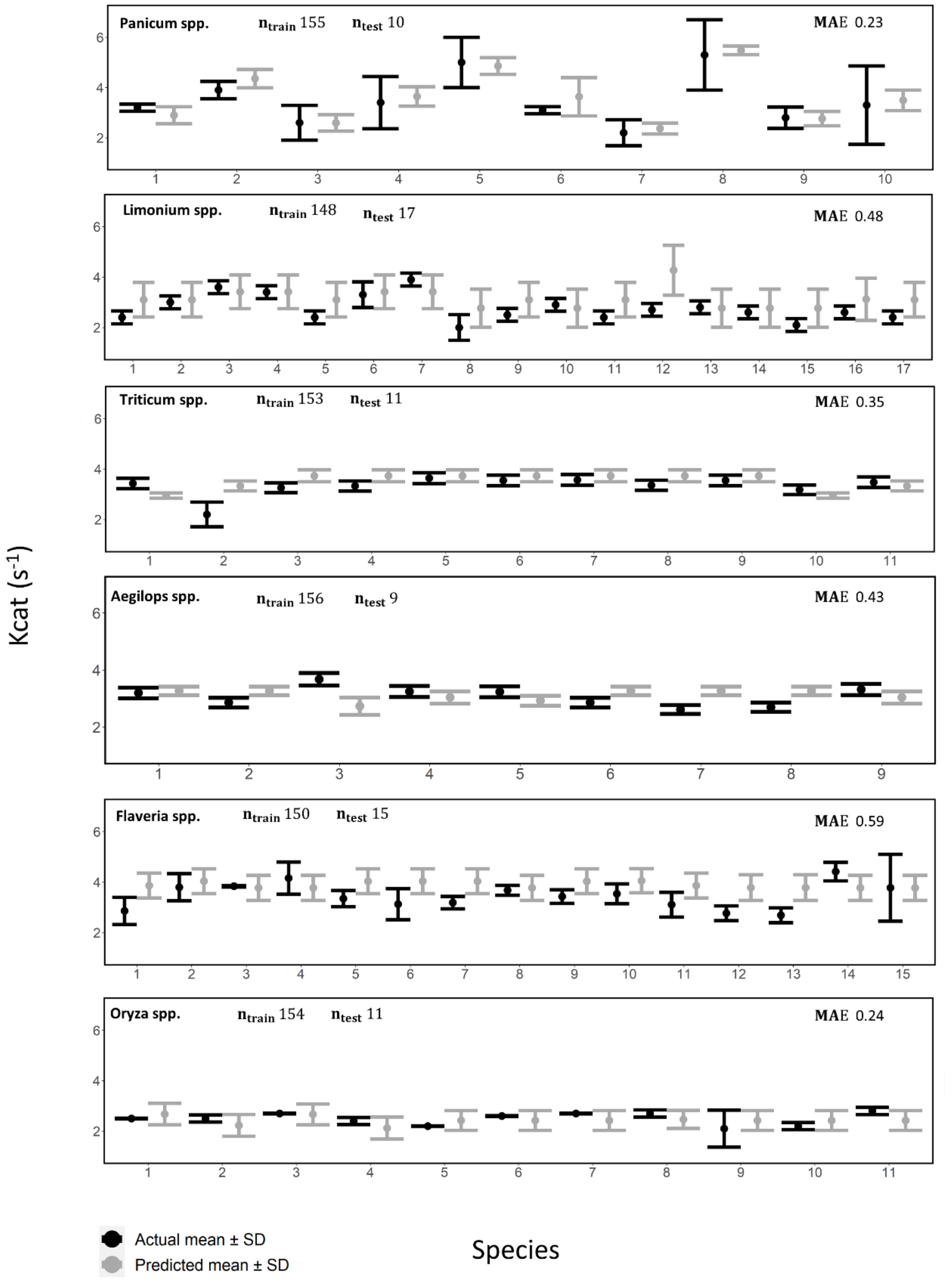

Figure S5. Leave-genus-out cross validation plots for carboxylation turnover rate (Kcat). Another validation framework that involved leaving a genus out during model training and then evaluating the model using the unseen genus. For example, Panicum spp. were removed during model training leaving 155 other species for training (n_{train}) and the model performance was assessed using the 10 unseen Panicum spp. (n_{test})**.** This process was repeated for each genus with more than one species. Each pair of results show the actual (black) and predicted (grey) mean Kcat ± SD. Actual or experimental SDs were calculated from the reported standard errors (SE) and number of replicates (see methods section).The mean absolute error (MAE) between the predicted and actual values are shown for each genus group.

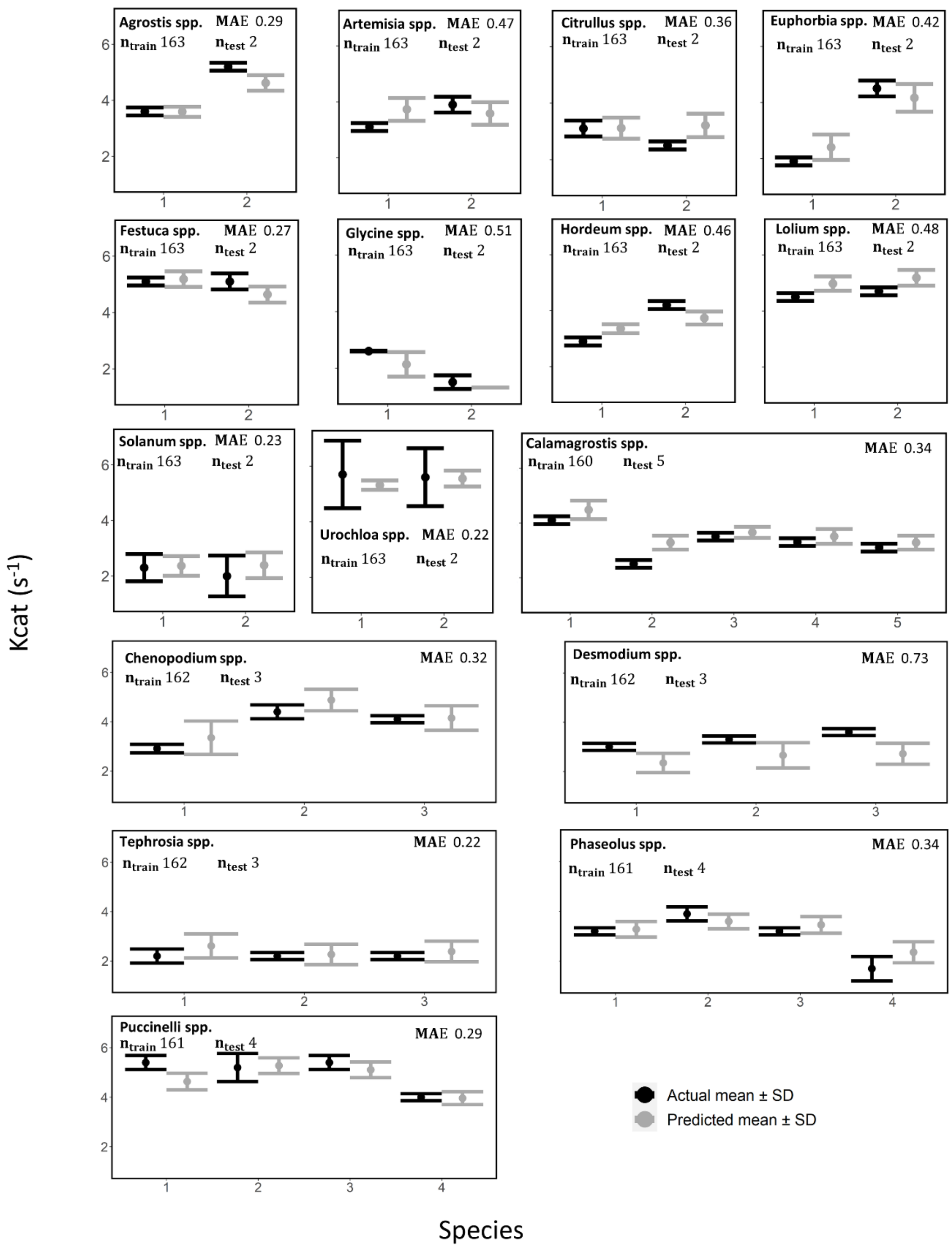

Figure S5 continued. Leave-genus-out cross validation plots for Kcat.

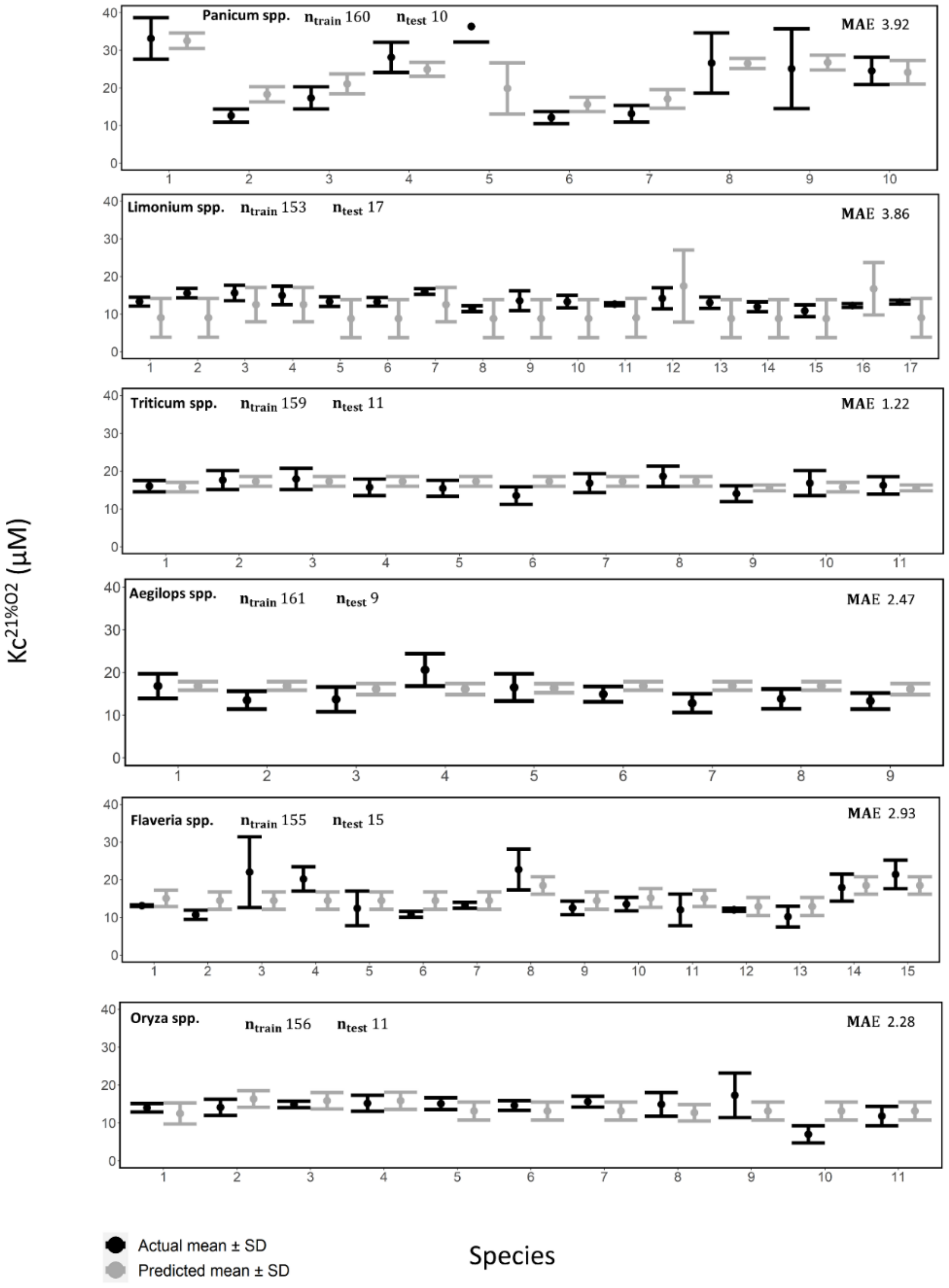

Figure S6. Leave-genus-out cross validation plots for the Michaelis-Menten constant for CO_2 at ambient atmospheric O_2 ($Kc^{21\%O2}$). Same process as figure S4. Each pair of results show the actual (black) and predicted (grey) mean $Kc^{21\%O2}$ ± SD. Actual or experimental SDs were calculated from the reported standard errors (SE) and number of replicates (see methods section). The mean absolute error (MAE) between the predicted and actual values are shown for each genus group.

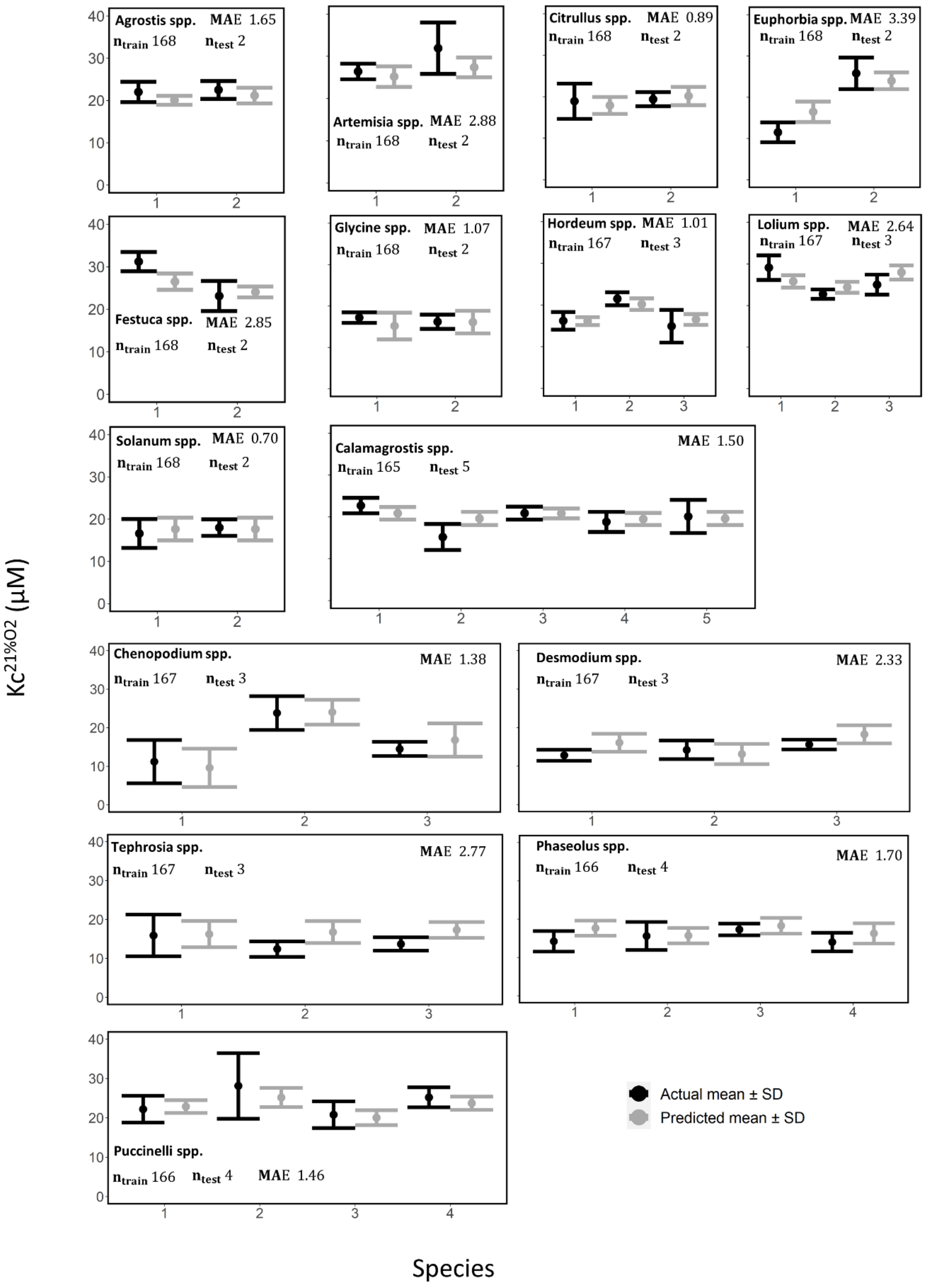

Figure S6 continued. Leave-genus-out cross validation plots for $Kc^{21\%O2}$.

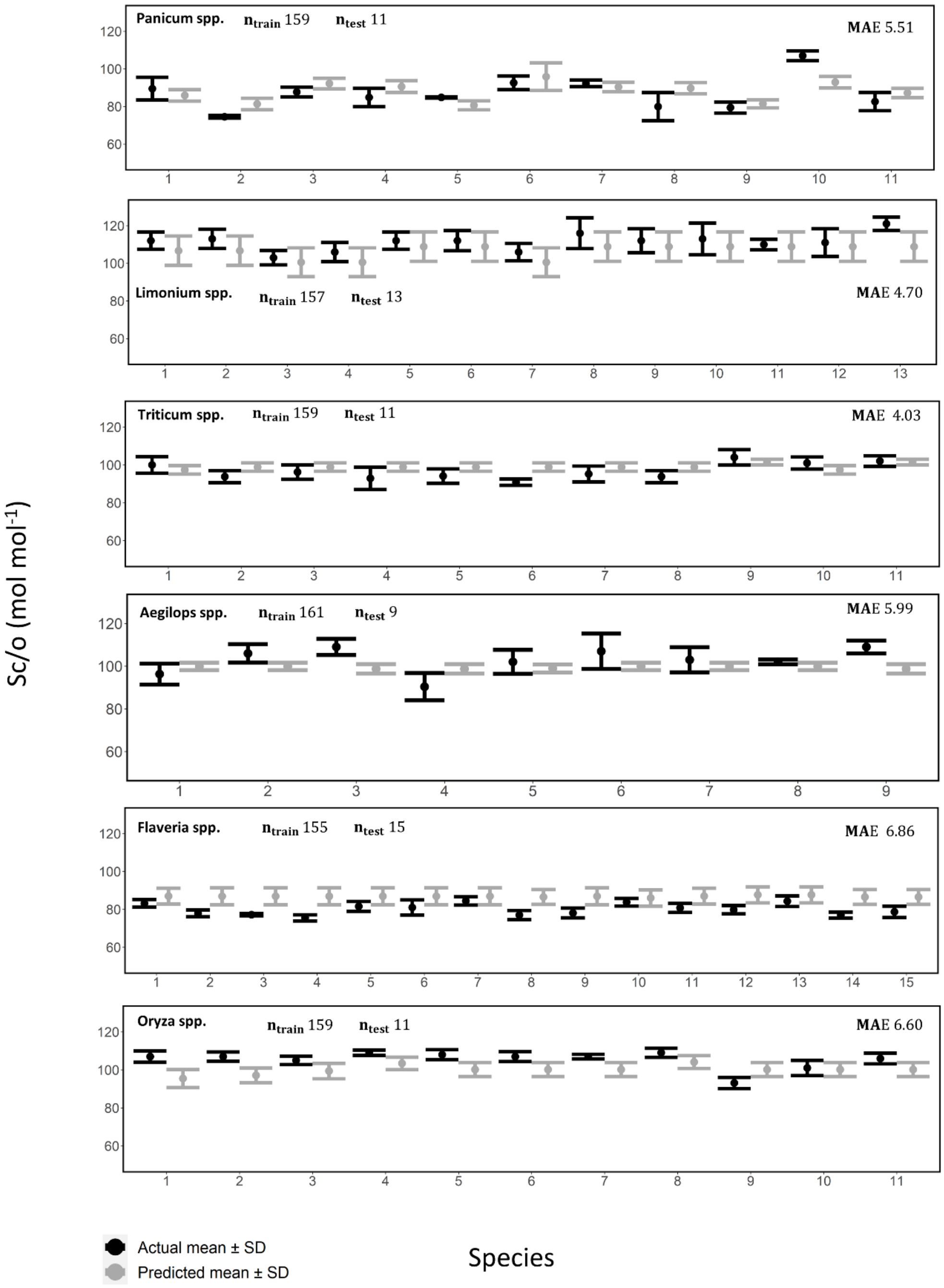

Figure S7. Leave-genus-out cross validation plots for the specificity for CO_2 over O_2 (Sc/o). Same process as figure S4. Each pair of results show the actual (black) and predicted (grey) mean Sc/o ± SD. Actual or experimental SDs were calculated from the reported standard errors (SE) and number of replicates (see methods section). The mean absolute error (MAE) between the predicted and actual values are shown for each genus group.

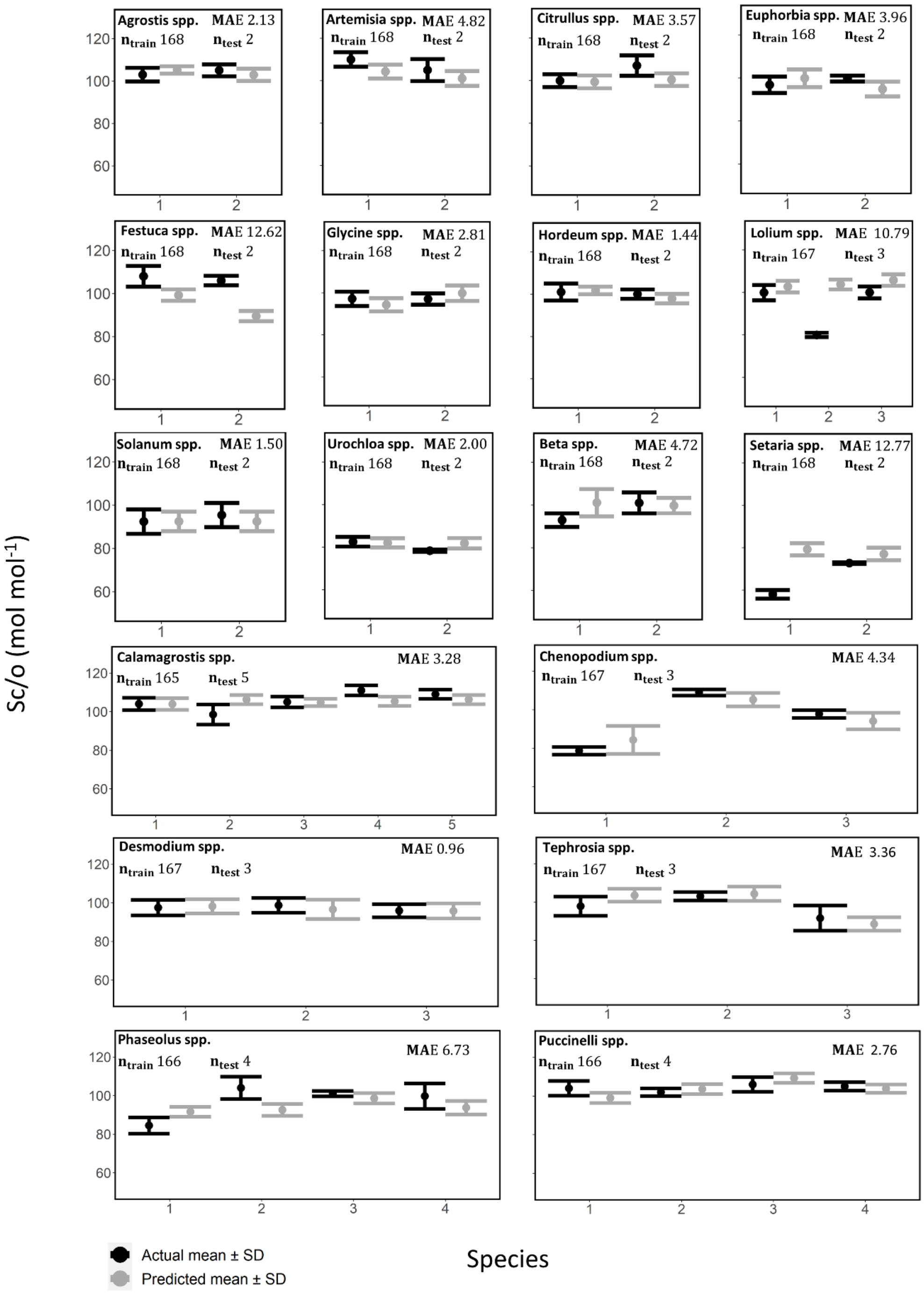

Figure S7. Continued. Leave-genus-out cross validation plots for Sc/o.

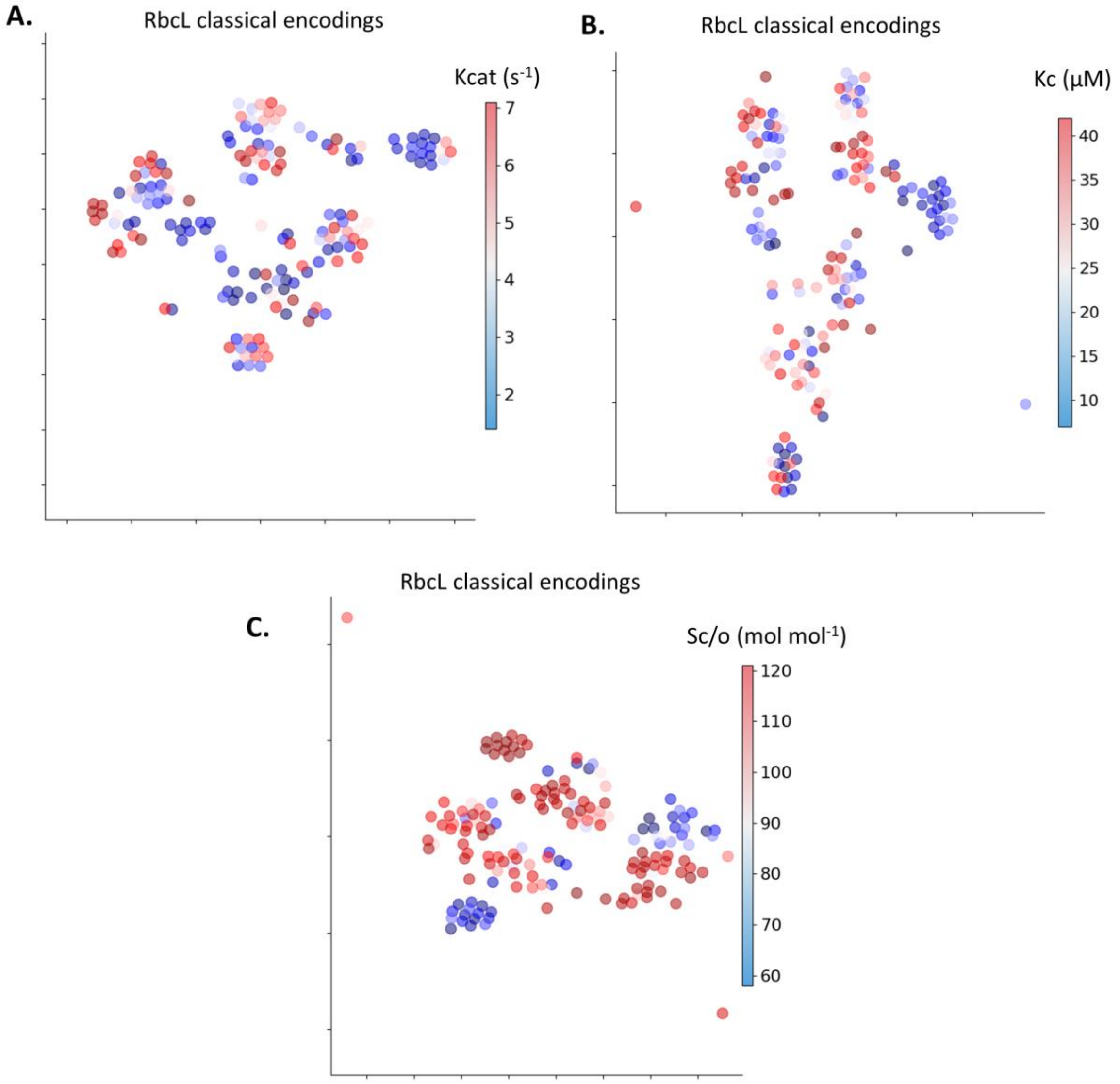

Figure S8. Visualization of the Rubisco large subunit (RbcL) classical encodings used in the fully trained Gaussian process (GP) models. Each data point represents an RbcL classical encoding with (A) carboxylation turnover rate (Kcat: s^{-1}) (n=165), (B) Michaelis-Menten constant for CO_2 at ambient atmospheric O_2 ($Kc^{21\%O2}$: µM) (n=170) and (C) specificity for CO_2 over O_2 (Sc/o: mol mol^{-1}) (n=170).

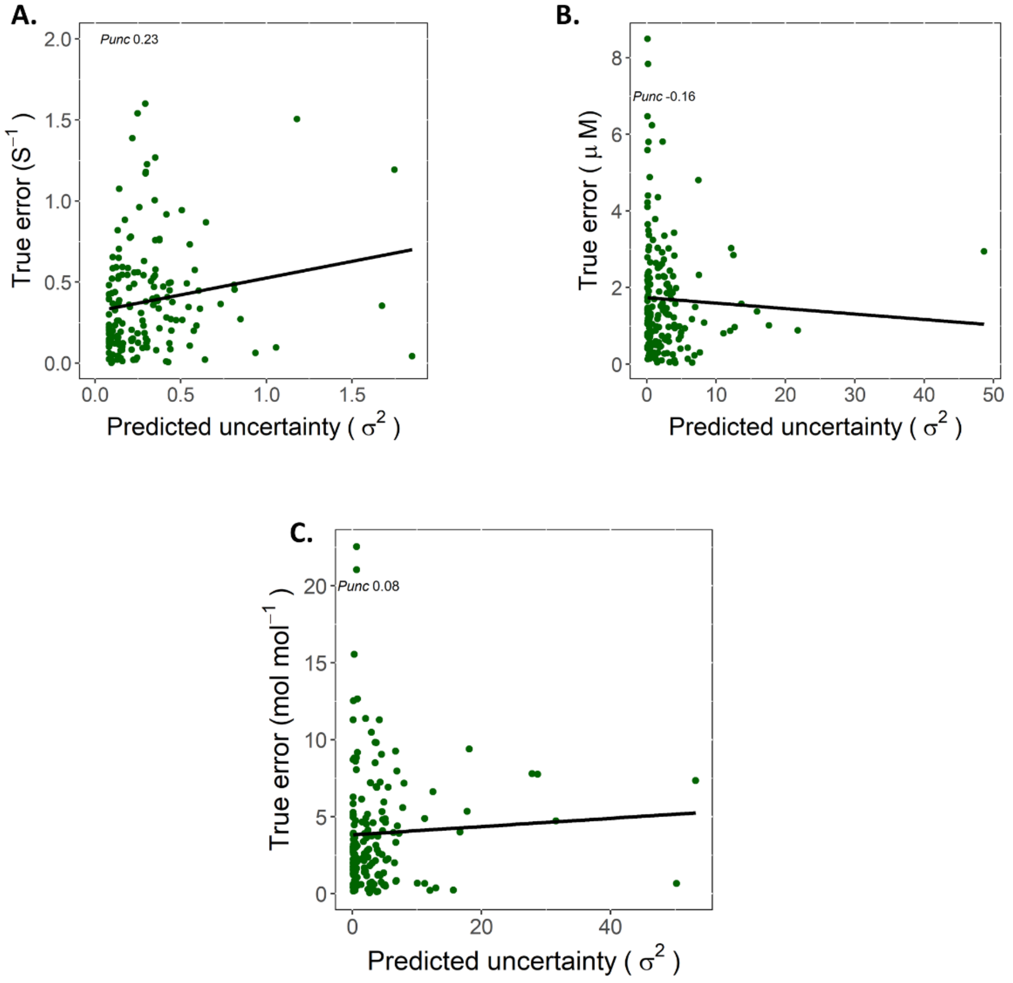

Figure S9. Spearman rank correlations (ρ_{unc}) of leave-one-out cross validation predicted uncertainties (σ^2) and true errors for (A) carboxylation turnover rate (Kcat : s^{-1}), (B) Michaelis-Menten constant for CO_2 at ambient O_2 ($Kc^{21\%O2}$: μM) and (C) specificity for CO_2 over O_2 (Sc/o: mol mol^{-1}). Determined using the learned encodings (Rives et al., 2021) encoded by an additive Matern 5/2 kernel.

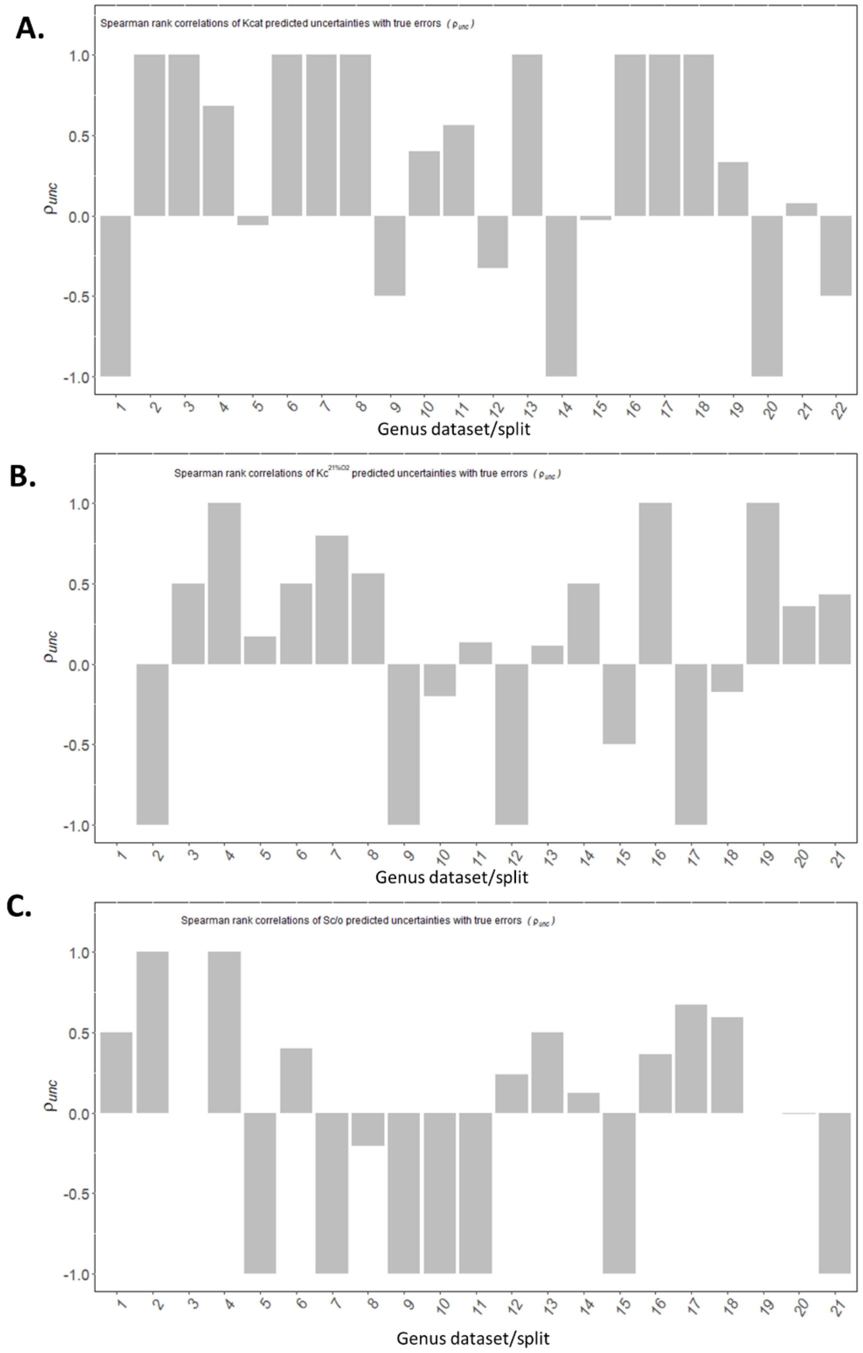

Figure S10. Spearman rank correlations (ρ_{unc}) of leave-genus-out cross validation predicted uncertainties (σ^2) and true errors for (A) carboxylation turnover rate (Kcat : s^{-1}), (B) Michaelis-Menten constant for CO_2 at ambient O_2 ($Kc^{21\%O2}$: μM) and (C) specificity for CO_2 over O_2 (Sc/o: mol mol^{-1}). Determined using the learned encodings (Rives et al., 2021) encoded by an additive Matern 5/2 kernel. ρ_{unc} values are ordered by the mean absolute error (MAE) for each genus group.

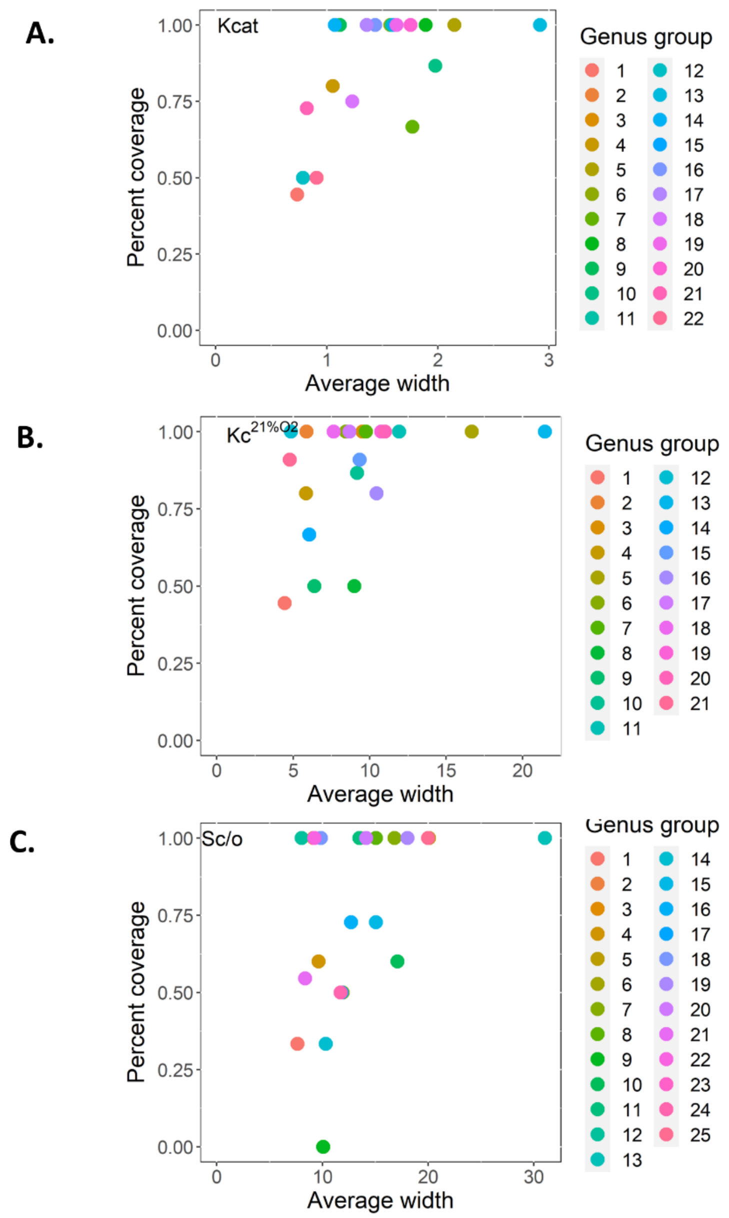

Figure S11. Coverage vs average width (4σ) for (A) carboxylation turnover rate (Kcat : s^{-1}), (B) Michaelis-Menten constant for CO_2 at ambient O_2 ($Kc^{21\%O2}$: μM) and (C) specificity for CO_2 over O_2 (Sc/o: mol mol^{-1}) leave-genus-out cross validation predictions. Coverage is the percentage of actual mean values that fall within the predicted 95% confidence intervals (±2σ). High coverage and low width indicates good model uncertainty. As predicted mean values become increasingly out of distribution, we would like width to increase indicating increasing model uncertainty and coverage to remain high.

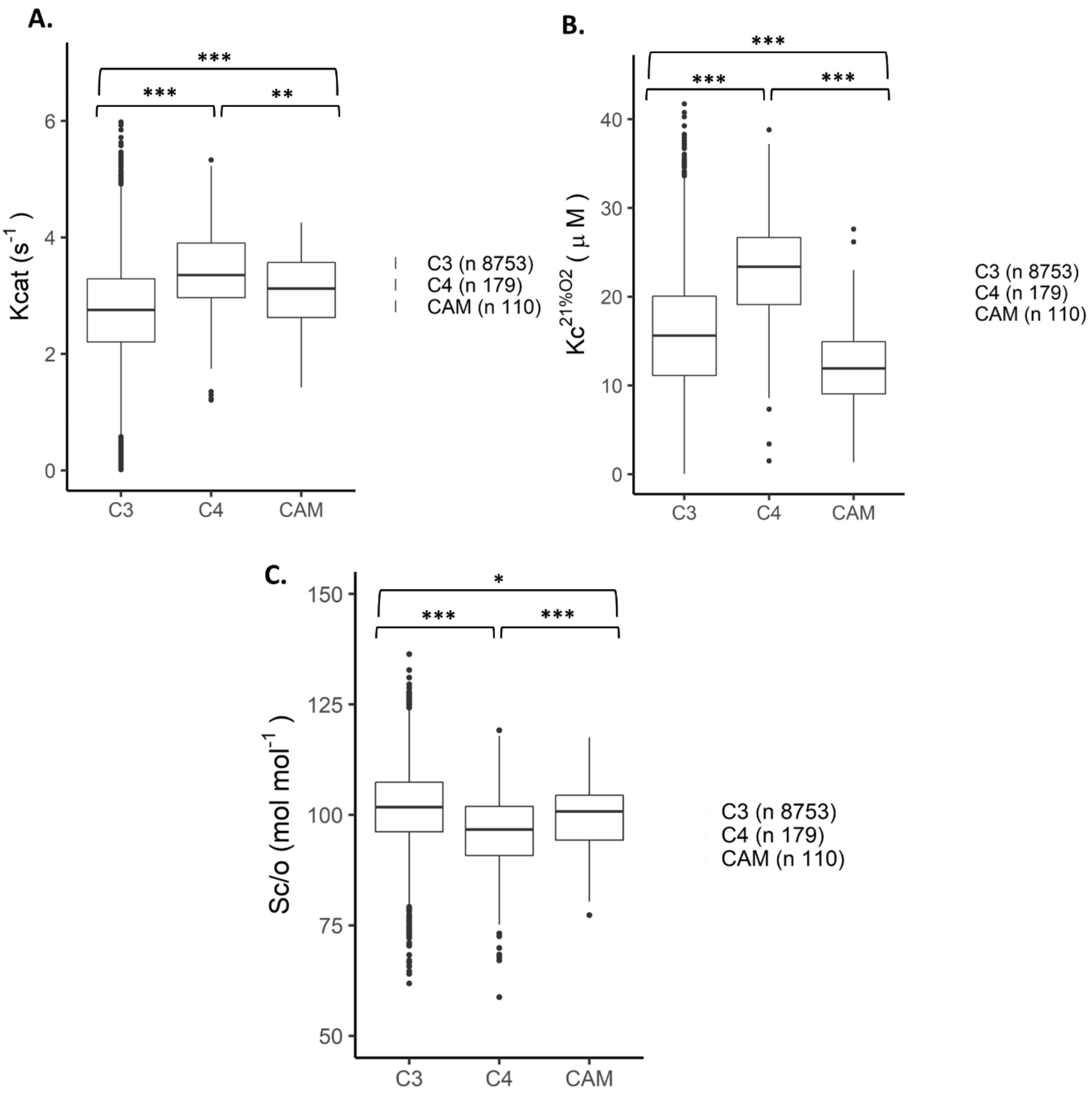

Figure S12. Box plots depict (A) carboxylation turnover rate (Kcat: s^{-1}), (B) Michaelis-Menten constant for CO_2 at ambient atmospheric O_2 ($Kc^{21\%O2}$: μM) and (C) specificity of CO_2 to O_2 (Sc/o: mol mol^{-1}) predictions made for the wild-type form IB (plants) Rubisco large subunit (RbcL) sequence-space using the fully trained Gaussian process (GP) models with the learned encoding scheme. Predictions are shown for all known land plant Rubiscos. Predictions were grouped by photosynthesis metabolism type (C_3, C_4 or CAM). Box plot horizontal lines show the median value, and the box and whisker represent the 25th and 75th percentile and minimum to maximum distributions of the data. Significant differences from the one-way ANOVA with Duncan's post hoc test are shown for groups: *** p<0.001, ** p<0.01, * p<0.05, n.s., non significant.

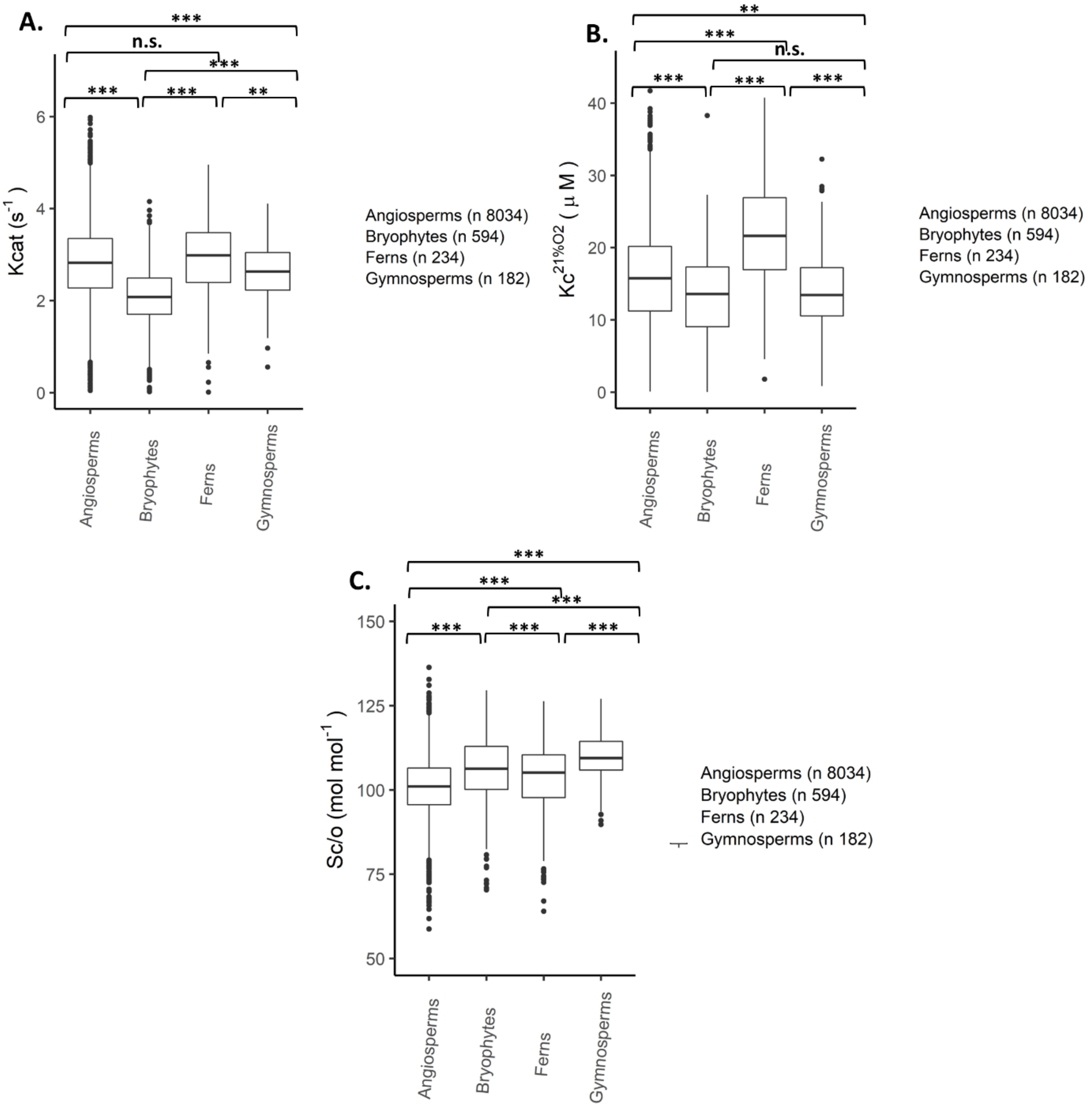

Figure S13. Box plots depict (A) carboxylation turnover rate (Kcat: s^{-1}), (B) Michaelis-Menten constant for CO_2 at ambient atmospheric O_2 (Kc$^{21\%O2}$: μM) and (C) specificity of CO_2 to O_2 (Sc/o: mol mol^{-1}) predictions made for the wild-type form IB (plants) Rubisco large subunit (RbcL) sequence-space using the fully trained Gaussian process (GP) models with the learned encoding scheme. Predictions are shown for all known land plant Rubiscos. Predictions were grouped by taxonomical type (Angiosperms, Ferns (including Pteridophytes and Lycopodiophytes) Gymnosperms or Bryophytes). Box plot horizontal lines show the median value, and the box and whisker represent the 25th and 75th percentile and minimum to maximum distributions of the data. Significant differences from the one-way ANOVA with Duncan's post hoc test are shown for groups: *** $p<0.001$, ** $p<0.01$, * $p<0.05$, n.s., non significant.

Table S2. Rubisco experimental kinetics and Rubisco large subunit (RbcL) sequences for training Gaussian process models.

Species	Accession No.	Photosynthetic type	Database	Kcat mean	Kcat SD	Sc/o mean	Sc/o SD	$Kc^{21\%O2}$ mean	$Kc^{21\%O2}$ SD	Citation
Aegilops biuncialis	LN626633	C_3 plant	EMBL	3.2	0.2	96.3	4.9	16.8	2.9	Prins et al. (2016)
Aegilops comosa	LN626632	C_3 plant	EMBL	2.9	0.2	106.0	4.3	13.5	2.1	Prins et al. (2016)
Aegilops cylindrica	LN626627	C_3 plant	EMBL	3.7	0.2	109.0	3.8	13.7	2.9	Prins et al. (2016)
Aegilops juvenalis	CEH24677.1	C_3 plant	NCBI	3.3	0.2	90.4	6.4	20.6	3.8	Prins et al. (2016)
Aegilops speltoides	LN626629	C_3 plant	EMBL	3.2	0.2	102.0	5.6	16.5	3.2	Prins et al. (2016)
Aegilops tauschii	LN626630	C_3 plant	EMBL	2.9	0.2	107.0	8.3	14.9	1.8	Prins et al. (2016)
Aegilops triuncialis	LN626634	C_3 plant	EMBL	2.6	0.2	103.0	5.9	12.8	2.2	Prins et al. (2016)
Aegilops uniaristata	LN626631	C_3 plant	EMBL	2.7	0.2	102.0	1.1	13.8	2.3	Prins et al. (2016)
Aegilops vavilovii	LN626635	C_3 plant	EMBL	3.3	0.2	109.0	3.0	13.3	1.9	Prins et al. (2016)
Agriophyllum squarrosum	A0A1C3HPL7	C_3 plant	uni	2.8	0.1	93.9	1.1	15.4	1.5	Orr et al. (2016)
Agrostis scabra	A0A1C3HPM5	C_3 plant	uni	3.6	0.1	103.0	1.6	22.0	1.7	Orr et al. (2016)
Agrostis stolonifera	ABK79588.1	C_3 plant	NCBI	5.2	0.1	105.0	1.4	25.3	1.5	Orr et al. (2016)
Amaranthus hybridus	YP_010028517.1	C_4 plant	NCBI	3.8	0.3	82.0	4.0	21.3	1.5	Savir et al. (2010)
Amphicarpaea bracteata	A0A1C3HPM0	C_3 plant	uni	4.0	0.1	97.5	2.2	29.0	2.1	Orr et al. (2016)
Arabidopsis thaliana	QDK58872.1	C_3 plant	NCBI	4.1	0.3	105.0	7.3	16.1	0.7	Galmés et al. (2014b)
Arctagrostis latifolia	A0A1C3HPM4	C_3 plant	uni	5.8	0.1	105.0	1.4	21.0	1.1	Orr et al. (2016)
Artemisia myriantha	A0A1C3HPM2	C_3 plant	uni	3.1	0.1	110.0	1.7	26.4	1.3	Orr et al. (2016)
Artemisia vulgaris	LT576797	C_3 plant	EMBL	3.9	0.2	105.0	2.6	31.9	4.3	Orr et al. (2016)
Avena sativa	AAA84028.1	C_3 plant	NCBI	2.3	0.3	99.9	3.0	18.1	2.0	Hermida-Carrera et al. (2016)
Beta maritima ssp. maritima	AEK33913.1	C_3 plant	NCBI			92.9	3.2			Galmes et al. (2005)
Beta vulgaris	AAY68360.1	C_3 plant	NCBI	2.0	0.3	101.0	2.0	18.6	1.1	Hermida-Carrera et al. (2016)
Brachypodium distachyon	LN626640	C_3 plant	EMBL	2.1	0.1	111.0	4.0	11.9	2.5	Prins et al. (2016)
Brassica oleracea	YP_009564583.1	C_3 plant	NCBI	2.1	0.3	96.2	1.3	19.2	0.3	Hermida-Carrera et al. (2016)
Bromus anomalus	A0A1C3HPM1	C_3 plant	uni	2.9	0.2	101.0	3.2	16.9	3.1	Orr et al. (2016)

Calamagrostis arundinacea	A0A1C3HPL9	C_3 plant	uni	4.1	0.1	104.0	1.6	22.7	1.3	Orr et al. (2016)
Calamagrostis canescens	A0A1C3HPN2	C_3 plant	uni	2.5	0.1	98.5	2.6	15.2	2.2	Orr et al. (2016)
Calamagrostis foliosa	A0A1C3HPN4	C_3 plant	uni	3.5	0.1	105.0	1.4	20.9	1.1	Orr et al. (2016)
Calamagrostis inexpansa	A0A1C3HPM7	C_3 plant	uni	3.3	0.1	111.0	1.3	18.8	1.7	Orr et al. (2016)
Calamagrostis nutkaensis	A0A1C3HPN0	C_3 plant	uni	3.1	0.1	109.0	1.2	20.1	2.8	Orr et al. (2016)
Capsicum annuum	A0A1U8QD66	C_3 plant	uni	1.9	0.1	96.0	4.5	19.8	1.5	Hermida-Carrera et al. (2016)
Cenchrus ciliaris	YP_009573569.1	C_4 plant	NCBI	6.0	0.8	69.9	3.0	29.2	2.3	Sharwood et al. (2016)
Chenopodium album	ATL76552.1	C_3 plant	NCBI	2.9	0.1	78.7	1.0	11.2	2.8	Kubien et al. (2008)
Chenopodium murale	SBO07486.1	C_3 plant	NCBI	4.4	0.2	109.0	0.8	23.8	3.1	Orr et al. (2016)
Chenopodium rubrum	A0A1C3HPN3	C_3 plant	uni	4.1	0.1	97.8	1.0	14.5	1.3	Orr et al. (2016)
Chrysanthellum indicum var. afroamericanum	A0A1C3HPM6	C_4 plant	uni	4.7	0.1	82.4	0.6	28.1	2.3	Orr et al. (2016)
Citrullus ecirrhosus	A0A1C3HPM8	C_3 plant	uni	3.1	0.2	99.9	1.5	18.9	3.0	Orr et al. (2016)
Citrullus lanatus var. lanatus	SBO07491.1	C_3 plant	NCBI	2.5	0.1	107.0	2.4	19.4	1.2	Orr et al. (2016)
Coffee arabica	YP_817490.1	C_3 plant	NCBI	2.1	0.2	98.7	3.8	22.9	2.4	Hermida-Carrera et al. (2016)
Crithmum maritimum	YP_004733335.1	C_3 plant	NCBI	3.4	0.1			18.7	0.2	Galmés et al. (2014b)
Cucurbita maxima	YP_009447458.1	C_3 plant	NCBI	2.2	0.2	98.4	0.4	19.2	1.0	Hermida-Carrera et al. (2016)
Cynodon dactylon	QYC94651.1	C_4 plant	NCBI			89.2	9.0	32.0	2.0	Carmo-Silva et al. (2010)
Dactylis glomerata cv. Porto	AJV89540.1	C_3 plant	NCBI	3.2	0.2			15.7	0.1	Galmés et al. (2014b)
Deschampsia danthonioides	A0A1C3HPP5	C_3 plant	uni	4.5	0.1	108.0	1.6	22.3	1.4	Orr et al. (2016)
Desmodium cinereum	A0A1C3HPP3	C_3 plant	uni	3.0	0.1	97.5	2.0	12.8	1.0	Orr et al. (2016)
Desmodium intortum	A0A1C3HPN7	C_3 plant	uni	3.3	0.1	98.7	1.9	14.2	1.7	Orr et al. (2016)
Desmodium psilocarpum	A0A1C3HPP4	C_3 plant	uni	3.6	0.1	95.9	1.7	15.6	0.9	Orr et al. (2016)
Echinochloa crus-galli	QPB15306.1	C_4 plant	NCBI			83.0	3.0			Jordan and Ogren (1983)

Elymus farctus	A0A1C3HPN9	C_3 plant	uni	3.3	0.1	106.0	1.3	19.5	2.7	Orr et al. (2016)
Eragrostis tef	A0A6C0SV93	C_4 plant	uni	7.1	0.2	89.0	2.1	34.9	2.1	Orr et al. (2016)
Erythrina flabelliformis	A0A1C3HPP1	C_3 plant	uni	3.6	0.1	96.4	1.9	18.4	1.9	Orr et al. (2016)
Espeletia schultzii	AIL52300.1	C_3 plant	NCBI					21.7	2.0	Castrillo (1995)
Euphorbia helioscopia	A0A6M4AEZ2	C_3 plant	uni	1.9	0.1	96.8	1.9	11.5	1.7	Orr et al. (2016)
Euphorbia microsphaera	A0A1C3HPP0	C_3 plant	uni	4.5	0.2	99.7	0.7	25.8	2.7	Orr et al. (2016)
Festuca gigantea	A0A1C3HPN8	C_3 plant	uni	5.1	0.1	108.0	2.4	31.2	1.6	Orr et al. (2016)
Festuca pratensis	K4PCJ4	C_3 plant	uni	5.1	0.2	106.0	1.1	23.1	2.5	Orr et al. (2016)
Flaveria angustifolia	ADW80651.1	C_3-C_4 plant	NCBI	2.9	0.2	83.2	1.0	13.1	0.1	Kubien et al. (2008)
Flaveria anomala	ADW80650.1	C_3-C_4 plant	NCBI	3.8	0.2	77.9	0.9	10.7	0.6	Kubien et al. (2008)
Flaveria australasica	P19161.1	C_4 plant	NCBI	3.8	0.0	77.2	0.3	22.0	4.7	Kubien et al. (2008)
Flaveria bidentis	P19161.1	C_4 plant	NCBI	4.2	0.3	75.5	0.8	20.2	1.6	Kubien et al. (2008)
Flaveria chloraefolia	ADW80653.1	C_3-C_4 plant	NCBI	3.4	0.1	81.6	1.3	12.4	2.3	Kubien et al. (2008)
Flaveria cronquistii	ADW80654.1	C_3 plant	NCBI	3.1	0.3	81.0	2.0	10.8	0.4	Kubien et al. (2008)
Flaveria floridana	ADW80655.1	C_3-C_4 plant	NCBI	3.2	0.1	84.5	1.1	13.2	0.4	Kubien et al. (2008)
Flaveria kochiana	ADW80656.1	C_4 plant	NCBI	3.7	0.1	77.0	1.2	22.7	2.7	Kubien et al. (2008)
Flaveria linearis	ADW80657.1	C_3-C_4 plant	NCBI	3.4	0.1	78.1	1.3	12.5	0.9	Kubien et al. (2008)
Flaveria palmeri	ADW80658.1	C_4 plant	NCBI	3.5	0.2	83.8	1.0	13.5	0.9	Kubien et al. (2008)
Flaveria pringlei	ADW80648.1	C_3 plant	NCBI	3.1	0.2	80.8	1.2	12.0	2.1	Kubien et al. (2008)
Flaveria ramosissima	ADW80659.1	C_3-C_4 plant	NCBI	2.8	0.1	79.8	1.1	12.0	0.2	Kubien et al. (2008)
Flaveria sonorensis	ADW80660.1	C_3-C_4 plant	NCBI	2.7	0.1	84.3	1.4	10.2	1.4	Kubien et al. (2008)
Flaveria trinervia	ADW80661.1	C_4 plant	NCBI	4.4	0.2	77.0	0.8	17.9	1.8	Kubien et al. (2008)
Flaveria vaginata	ADW80662.1	C_4 plant	NCBI	3.8	0.5	78.7	1.5	21.4	1.9	Kubien et al. (2008)
Flueggea suffruticosa	AFU96118.1	C_3 plant	NCBI	3.4	0.1	101.0	1.5	19.2	1.5	Orr et al. (2016)
Foeniculum vulgare	AMD83923.1	C_3 plant	NCBI	4.4	0.2	94.3	2.7	20.7	2.7	Orr et al. (2016)
Glycine canescens	YP_008145856.1	C_3 plant	NCBI	2.6	0.0	97.1	1.7	17.2	0.9	Orr et al. (2016)
Glycine max	ABC25107.1	C_3 plant	NCBI	1.5	0.1	97.0	1.1	16.2	0.7	Hermida-Carrera et al. (2016)
Helianthus annuus L.	XP_035835866.1	C_3 plant	NCBI			73.6	18.3			Viil et al. (2012)
Hordeum brachyantherum	A0A1C3HPQ0	C_3 plant	uni	2.9	0.1	101.0	2.0	16.2	1.5	Orr et al. (2016)
Hordeum murinum	A0A1C3HPQ4	C_3 plant	uni	4.2	0.1	100.0	1.1	21.5	1.1	Orr et al. (2016)

Hordeum vulgare ssp. vulgare cv. Morex	ASD42799.1	C_3 plant	NCBI					14.9	1.6	Hermida-Carrera et al. (2016)
Ipomoea batatas	AFU50388.1	C_3 plant	NCBI	2.5	0.1	98.5	6.6	21.1	1.0	Hermida-Carrera et al. (2016)
Iris douglasiana	BAO57024.1	C_3 plant	NCBI	3.5	0.2			14.6	0.9	Galmés et al. (2014b)
Lablab purpureus	YP_010046451.1	C_3 plant	NCBI	5.3	0.1	91.1	1.7	21.7	1.6	Orr et al. (2016)
Lactuca sativa	YP_398337.1	C_3 plant	NCBI			94.0	1.9	18.2	1.4	Hermida-Carrera et al. (2016)
Lepidium campestre	A0A1C3HPQ1	C_3 plant	uni	3.4	0.1	92.8	1.2	15.8	1.0	Orr et al. (2016)
Limonium antonii-llorensii	AID50137.1	C_3 plant	NCBI	2.4	0.1	112.0	1.8	13.3	0.5	Galmés et al. (2014a)
Limonium artruchium	AID50126.1	C_3 plant	NCBI	3.0	0.1	113.0	2.0	15.5	0.5	Galmés et al. (2014a)
Limonium balearicum	AID50147.1	C_3 plant	NCBI	3.6	0.1	103.0	1.5	15.6	0.8	Galmés et al. (2014a)
Limonium barceloi	AID50128.1	C_3 plant	NCBI	3.4	0.1	106.0	2.0	14.9	1.0	Galmés et al. (2014a)
Limonium biflorum	AID50121.1	C_3 plant	NCBI	2.4	0.1	112.0	1.8	13.3	0.5	Galmés et al. (2014a)
Limonium companyonis	AID50113.1	C_3 plant	NCBI	3.3	0.2	112.0	2.1	13.3	0.4	Galmés et al. (2014a)
Limonium echioides	AID50149.1	C_3 plant	NCBI	3.9	0.1	106.0	1.8	16.0	0.3	Galmés et al. (2014a)
Limonium ejulabilis	AID50114.1	C_3 plant	NCBI	2.0	0.2	116.0	3.2	11.4	0.3	Galmés et al. (2014a)
Limonium gibertii	AID50136.1	C_3 plant	NCBI	2.5	0.1	112.0	2.5	13.5	1.0	Galmés et al. (2014a)
Limonium grosii	AID50112.1	C_3 plant	NCBI	2.9	0.1	113.0	3.3	13.3	0.7	Galmés et al. (2014a)
Limonium gymnesicum	AID50143.1	C_3 plant	NCBI	2.4	0.1	121.0	2.3	12.6	0.2	Galmés et al. (2014a)
Limonium latebracteatum	BAO57039.1	C_3 plant	NCBI	2.7	0.1			14.2	1.6	Galmés et al. (2014b)
Limonium leonardi-llorensii	AID50109.1	C_3 plant	NCBI	2.8	0.1	110.0	1.1	13.0	0.6	Galmés et al. (2014a)
Limonium magallufianum	AID50120.1	C_3 plant	NCBI	2.6	0.1	111.0	2.9	11.9	0.5	Galmés et al. (2014a)
Limonium retusum	AID50105.1	C_3 plant	NCBI	2.1	0.1	121.0	1.4	10.9	0.6	Galmés et al. (2014a)
Limonium stenophyllum	BAO57038.1	C_3 plant	NCBI	2.6	0.1			12.3	0.3	Galmés et al. (2014b)
Limonium virgatum	AID50151.1	C_3 plant	NCBI	2.4	0.1			13.2	0.3	Galmés et al. (2014b)
Lolium multiflorum	AFV62913.1	C_3 plant	NCBI	4.5	0.1	99.9	1.8	29.1	2.1	Orr et al. (2016)
Lolium perenne	CAO85984.1	C_3 plant	NCBI			80.0	1.0	22.7	1.1	Savir et al. (2010)
Lolium rigidum	A0A1C3HPR5	C_3 plant	uni	4.7	0.1	100.0	1.4	25.0	1.7	Orr et al. (2016)

Macrotyloma uniflorum	ACJ38561.1	C_3 plant	NCBI	4.4	0.2	101.0	2.4	25.2	3.3	Orr et al. (2016)
Manihot esculenta	QFO47553.1	C_3 plant	NCBI	1.4	0.1	101.0	0.9	10.8	0.6	Hermida-Carrera et al. (2016)
Medicago sativa	A0A1B3T043	C_3 plant	uni	1.7	0.1	95.6	2.2	16.4	1.9	Hermida-Carrera et al. (2016)
Megathyrsus maximus	YP_009260243.1	C_4 plant	NCBI	5.3	0.5	80.3	2.8	27.1	1.1	Sharwood et al. (2016)
Mentha aquatica	ADU79049.1	C_3 plant	NCBI			97.2	3.3			Galmes et al. (2005)
Mercurialis annua	A0A1C3HPR2	C_3 plant	uni	3.4	0.2	95.7	2.7	17.0	3.7	Orr et al. (2016)
Musa velutina	UCC34423.1	C_3 plant	NCBI	3.2	0.1	111.0	1.8	19.0	1.9	Orr et al. (2016)
Nicotiana tabacum	1RLC_L	C_3 plant	NCBI	3.1	0.3	80.0	2.6	18.3	0.1	Long et al. (2018)
Oryza barthii/glaberrima	LT576837	C_3 plant	EMBL	2.5	0.0	107.0	1.5	14.0	0.8	Orr et al. (2016)
Oryza eichingeri	LT576838	C_3 plant	EMBL	2.5	0.1	107.0	1.2	14.1	1.5	Orr et al. (2016)
Oryza glaberrima (WAB 1939)	LT576839	C_3 plant	EMBL	2.7	0.0	105.0	1.1	14.9	0.6	Orr et al. (2016)
Oryza glumaepatula	LT576840	C_3 plant	EMBL	2.4	0.1	109.0	0.7	15.2	1.5	Orr et al. (2016)
Oryza longistaminata	LT576841	C_3 plant	EMBL	2.2	0.0	108.0	1.3	15.1	1.1	Orr et al. (2016)
Oryza meridionalis	LT576842	C_3 plant	EMBL	2.6	0.0	107.0	1.3	14.6	0.9	Orr et al. (2016)
Oryza nivara	LT576843	C_3 plant	EMBL	2.7	0.0	107.0	0.6	15.6	1.0	Orr et al. (2016)
Oryza punctata	LT576844	C_3 plant	EMBL	2.7	0.1	109.0	1.2	14.9	2.2	Orr et al. (2016)
Oryza sativa	ANG44656.1	C_3 plant	NCBI	2.1	0.3	93.1	1.2	17.3	2.4	Hermida-Carrera et al. (2016)
Oryza sativa subsp. Indica	LT576845	C_3 plant	EMBL	2.2	0.1	101.0	2.0	7.0	1.6	Orr et al. (2016)
oryza sativa subsp. Japonica	LT576846	C_3 plant	EMBL	2.8	0.1	106.0	1.4	11.8	1.8	Orr et al. (2016)
Pallenis maritima	BAO57025.1	C_3 plant	NCBI	2.7	0.1			10.6	0.5	Galmés et al. (2014b)
Panicum amarum	LT576849	C_4 plant	EMBL	3.2	0.1	89.5	3.0	33.1	3.9	Orr et al. (2016)
Panicum antidotale	SCM15150.1	C_4 plant	NCBI	3.9	0.2	74.5	0.4			Sharwood et al. (2016)
Panicum bisulcatum	SCM15147.1	C_3 plant	NCBI	2.6	0.4	87.7	1.5	12.6	1.0	Sharwood et al. (2016)
Panicum coloratum	SCM15156.1	C_4 plant	NCBI	3.4	0.6	84.8	2.8	17.3	1.7	Sharwood et al. (2016)
Panicum deustum	SCM15159.1	C_4 plant	NCBI	5.0	0.5	84.8	0.2	28.1	2.0	Sharwood et al. (2016)
Panicum dichotomiflorum	LT576847	C_4 plant	EMBL	3.1	0.1	92.6	1.8	36.3	2.9	Orr et al. (2016)

Panicum milioides	SCM15149.1	C_3 plant	NCBI	2.2	0.3	92.3	1.0	12.1	0.8	Sharwood et al. (2016)
Panicum milliaceum	SCM15155.1	C_4 plant	NCBI			79.9	4.3	13.1	1.1	Sharwood et al. (2016)
Panicum monticola	SCM15151.1	C_4 plant	NCBI	5.3	0.7	79.4	1.7	26.6	4.0	Sharwood et al. (2016)
Panicum phragmitoides	LT576848	C_4 plant	EMBL	2.8	0.3	107.0	1.3	25.1	7.5	Orr et al. (2016)
Panicum virgatum	SCM15154.1	C_4 plant	NCBI	3.3	0.9	82.6	2.8	24.5	2.1	Sharwood et al. (2016)
Paspalum dilatatum	YP_009267237.1	C_4 plant	NCBI			88.0	7.1	30.0	1.2	Carmo-Silva et al. (2010)
Petroselinum cripsum	YP_004733851.1	C_3 plant	NCBI			77.0	2.0			Jordan and Ogren (1983)
Phaseolus carteri	LT576850	C_3 plant	EMBL	3.2	0.1	84.5	2.1	14.2	1.9	Orr et al. (2016)
Phaseolus coccineus	LT576851	C_3 plant	EMBL	3.9	0.2	104.0	2.9	15.6	2.6	Orr et al. (2016)
Phaseolus lunatus	LT576852	C_3 plant	EMBL	3.2	0.1	101.0	0.7	17.3	1.1	Orr et al. (2016)
Phaseolus vulgaris	QCQ20610.1	C_3 plant	NCBI	1.7	0.2	99.7	2.7	14.0	1.0	Hermida-Carrera et al. (2016)
Pisum sativum	AIK21409.1	C_3 plant	NCBI			90.2	1.7			Uemura et al. (1997)
Plantago lanceolata L.	AAA84554.1	C_3 plant	NCBI			77.3	12.9			Viil et al. (2012)
Poa palustris	LT576854	C_3 plant	EMBL	4.2	0.1	112.0	2.1	19.2	1.3	Orr et al. (2016)
Portulaca oleracea	AFB70630.1	C_4 plant	NCBI	5.9	0.4	78.0	4.0			Savir et al. (2010)
Puccinellia distans	LT576855	C_3 plant	EMBL	5.4	0.2	104.0	1.9	22.2	2.4	Orr et al. (2016)
Puccinellia lemmonii	LT576856	C_3 plant	EMBL	5.2	0.4	102.0	1.0	28.1	5.9	Orr et al. (2016)
Puccinellia maritima	LT576857	C_3 plant	EMBL	5.4	0.2	106.0	1.9	20.8	2.4	Orr et al. (2016)
Puccinellia nuttalliana	LT576858	C_3 plant	EMBL	4.0	0.1	105.0	1.1	25.2	1.8	Orr et al. (2016)
Pueraria montana	QRM91166.1	C_3 plant	NCBI	2.7	0.1	101.0	1.0	20.9	1.6	Orr et al. (2016)
Saccharum officinarum	ASV52260.1	C_4 plant	NCBI	3.9	0.3	82.2	1.8	31.7	2.1	Hermida-Carrera et al. (2016)
Secale cereale	LN626639	C_3 plant	EMBL	3.2	0.2	91.5	4.1	20.2	2.1	Prins et al. (2016)
Setaria italica	AGZ13153.1	C_4 plant	NCBI			58.0	2.0			Jordan and Ogren (1983)
Setaria viridis	AGT56139.1	C_4 plant	NCBI	5.9	0.5	72.7	0.2	25.5	2.0	Sharwood et al. (2016)
Sideritis cretica subsp. spicata	BAO57028.1	C_3 plant	NCBI	2.0	0.3			12.8	0.2	Galmés et al. (2014b)
Solanum lycopersicum	ANW81480.1	C_3 plant	NCBI	2.3	0.2	92.4	2.3	16.6	1.4	Hermida-Carrera et al. (2016)
Solanum tuberosum	ABD47065.1	C_3 plant	NCBI	2.0	0.3	95.4	2.3	18.0	0.8	Hermida-Carrera et al. (2016)
Sorghum bicolor	YP_899415.1	C_4 plant	NCBI	5.4	0.1	70.0	1.0			Savir et al. (2010)

Sphenostylis stenocarpa	LT576860	C_3 plant	EMBL	2.8	0.1	93.6	3.4	17.4	1.5	Orr et al. (2016)
Spinacia oleracea	CAA23473.1	C_3 plant	NCBI	2.4	0.1	97.0	1.2	26.9	0.8	Hermida-Carrera et al. (2016)
Steinchisma laxa	YP_009261073.1	C_3 plant	NCBI	2.3	0.3	91.4	4.8			Sharwood et al. (2016)
Tephrosia candida	LT576861	C_3 plant	EMBL	2.2	0.2	97.8	2.5	15.9	3.8	Orr et al. (2016)
Tephrosia purpurea	LT576862	C_3 plant	EMBL	2.2	0.1	103.0	1.1	12.4	1.4	Orr et al. (2016)
Tephrosia rhodesica	LT576863	C_3 plant	EMBL	2.2	0.1	91.6	3.3	13.7	1.2	Orr et al. (2016)
Tetragonia expansa	YP_009462736.1	C_3 plant	NCBI			81.0	1.0	17.6	1.2	Savir et al. (2010)
Teucrium heterophyllum	BAO57027.1	C_3 plant	NCBI	2.7	0.1			10.6	0.2	Galmés et al. (2014b)
Trachycarpus fortunei	YP_010010626.1	C_3 plant	NCBI	2.8	0.1			14.2	1.3	Galmés et al. (2014b)
Trifolium repens	QGV12450.1	C_3 plant	NCBI					20.8	1.2	Lehnherr et al. (1985)
Triticale	LN626638	C_3 plant	EMBL	3.6	0.2	99.7	5.1	16.1	2.4	Prins et al. (2016)
Triticum aestivum	BCU02136.1	C_3 plant	NCBI	2.2	0.2	100.0	1.8	16.0	0.6	Hermida-Carrera et al. (2016)
Triticum aestivum SATYN1	LN626617	C_3 plant	EMBL	3.3	0.2	93.8	3.2	17.6	2.5	Prins et al. (2016)
Triticum aestivum SATYN2	LN626618	C_3 plant	EMBL	3.3	0.2	96.2	3.8	17.9	2.8	Prins et al. (2016)
Triticum aestivum SATYN3	LN626619	C_3 plant	EMBL	3.6	0.2	92.9	5.9	15.7	2.2	Prins et al. (2016)
Triticum dicoccon1	LN626623	C_3 plant	EMBL	3.6	0.2	94.1	3.8	15.4	2.1	Prins et al. (2016)
Triticum dicoccon2	LN626624	C_3 plant	EMBL	3.6	0.2	90.9	1.7	13.5	2.3	Prins et al. (2016)
Triticum dicoccon3	LN626625	C_3 plant	EMBL	3.4	0.2	95.2	4.2	16.8	2.5	Prins et al. (2016)
Triticum dicoccon4	LN626626	C_3 plant	EMBL	3.6	0.2	93.8	3.2	18.6	2.7	Prins et al. (2016)
Triticum monococcum	LN626620	C_3 plant	EMBL	3.2	0.2	104.0	4.1	14.0	2.1	Prins et al. (2016)
Triticum timonovum	LN626622	C_3 plant	EMBL	3.5	0.2	101.0	3.2	16.8	3.3	Prins et al. (2016)
Triticum timopheevii	LN626621	C_3 plant	EMBL	3.4	0.2	102.0	2.8	16.2	2.3	Prins et al. (2016)
Urochloa mosambicensis	SCM15160.1	C_4 plant	NCBI	5.7	0.7	82.5	1.3			Sharwood et al. (2016)
Urochloa panicoides	SCM15158.1	C_4 plant	NCBI	5.6	0.6	78.3	0.3	24.1	1.0	Sharwood et al. (2016)
Zea mays cv. Carella	CAA78027.1	C_4 plant	NCBI	4.1	0.6	87.3	1.4	42.0	2.8	Hermida-Carrera et al. (2016)
Zostera japonica	BAD05105.1	C_4 plant	NCBI			84.1	7.7	28.1	1.8	Carmo-Silva et al. (2010)

Carmo-Silva, A. E., Keys, A. J., Andralojc, P. J., Powers, S. J., Arrabaça, M. C. & Parry, M. A. 2010. Rubisco activities, properties, and regulation in three different C4 grasses under drought. *Journal of Experimental Botany,* 61**,** 2355-66.

Castrillo, M. 1995. Ribulose-1,5-bis-phosphate carboxylase activity in altitudinal populations of Espeletia schultzii Wedd. *Oecologia,* 101**,** 193-196.

Davidi, D., Shamshoum, M., Guo, Z., et al. 2020. Highly active rubiscos discovered by systematic interrogation of natural sequence diversity. *The EMBO Journal,* 39**,** e104081.

Galmés, J., Andralojc, P. J., Kapralov, M. V., Flexas, J., Keys, A. J., Molins, A., Parry, M. a. J. & Conesa, M. À. 2014a. Environmentally driven evolution of Rubisco and improved photosynthesis and growth within the C3 genus Limonium (Plumbaginaceae). *New Phytologist,* 203**,** 989-999.

Galmes, J., Flexas, J., Keys, A. J., Cifre, J., Mitchell, R. A., Madgwick, P. J., Haslam, R. P., Medrano, H. & Parry, M. A. 2005. Rubisco specificity factor tends to be larger in plant species from drier habitats and in species with persistent leaves. *Plant, Cell & Environment,* 28**,** 571-579.

Galmés, J., Kapralov, M. V., Andralojc, P. J., Conesa, M., Keys, A. J., Parry, M. A. & Flexas, J. 2014b. Expanding knowledge of the Rubisco kinetics variability in plant species: environmental and evolutionary trends. *Plant, Cell & Environment,* 37**,** 1989-2001.

Hermida-Carrera, C., Kapralov, M. V. & Galmés, J. 2016. Rubisco Catalytic Properties and Temperature Response in Crops. *Plant Physiology,* 171**,** 2549-2561.

Jordan, D. B. & Ogren, W. L. 1983. Species variation in kinetic properties of ribulose 1,5-bisphosphate carboxylase/oxygenase. *Archives of Biochemistry and Biophysics,* 227**,** 425-433.

Kubien, D. S., Whitney, S. M., Moore, P. V. & Jesson, L. K. 2008. The biochemistry of Rubisco in Flaveria. *Journal of Experimental Botany,* 59**,** 1767-1777.

Lehnherr, B., Mächler, F. & Nösberger, J. 1985. Influence of Temperature on the Ratio of Ribulose Bisphosphate Carboxylase to Oxygenase Activities and on the Ratio of Photosynthesis to Photorespiration of Leaves. *Journal of Experimental Botany,* 36**,** 1117-1125.

Long, B. M., Hee, W. Y., Sharwood, R. E., et al. 2018. Carboxysome encapsulation of the CO2-fixing enzyme Rubisco in tobacco chloroplasts. *Nature Communications,* 9**,** 3570.

Orr, D. J., Alcântara, A., Kapralov, M. V., Andralojc, P. J., Carmo-Silva, E. & Parry, M. a. J. 2016. Surveying Rubisco Diversity and Temperature Response to Improve Crop Photosynthetic Efficiency. *Plant Physiology,* 172**,** 707-717.

Prins, A., Orr, D. J., Andralojc, P. J., Reynolds, M. P., Carmo-Silva, E. & Parry, M. A. 2016. Rubisco catalytic properties of wild and domesticated relatives provide scope for improving wheat photosynthesis. *Journal of Experimental Botany,* 67**,** 1827-38.

Savir, Y., Noor, E., Milo, R. & Tlusty, T. 2010. Cross-species analysis traces adaptation of Rubisco toward optimality in a low-dimensional landscape. *Proceedings of the National Academy of Sciences,* 107**,** 3475-3480.

Sharwood, R. E., Ghannoum, O., Kapralov, M. V., Gunn, L. H. & Whitney, S. M. 2016. Temperature responses of Rubisco from Paniceae grasses provide opportunities for improving C3 photosynthesis. *Nature Plant,* 2**,** 16186.

Uemura, K., Anwaruzzaman, Miyachi, S. & Yokota, A. 1997. Ribulose-1,5-Bisphosphate Carboxylase/Oxygenase from Thermophilic Red Algae with a Strong Specificity for CO2Fixation. *Biochemical and Biophysical Research Communications,* 233**,** 568-571.

Viil, J., Ivanova, H. & Pärnik, T. 2012. Specificity factor of Rubisco: estimation in intact leaves by carboxylation at different CO2/O2 ratios. *Photosynthetica,* 50**,** 247-253.
